# CD5L promotes efferocytosis and resolution of retinal ischemic injury

**DOI:** 10.1038/s41419-026-08752-8

**Published:** 2026-04-20

**Authors:** Rami A. Shahror, Bushra Zaman, Piyanan Chuesiang, Melissa Wild, Esraa Shosha, Yuet-Kin Leung, Shengyu Mu, Nancy J. Rusch, Abdelrahman Y. Fouda

**Affiliations:** 1 Department of Pharmacology and Toxicology, College of Medicine, University of Arkansas for Medical Sciences, Little Rock, AR USA; 2 Clinical Pharmacy Department, Cairo University, Cairo, Egypt

**Keywords:** Neuro-vascular interactions, Translational research

## Abstract

Ischemia-induced retinopathy is a defining feature of prevalent ocular conditions, including diabetic retinopathy and central retinal artery or vein occlusion. Therapeutic interventions for ischemic retinopathies show limited efficacy and adverse effects, highlighting the need to thoroughly investigate the underlying mechanisms. Histone deacetylase 3 (HDAC3), a member of the histone deacetylase family, plays a central role in regulating gene expression in myeloid cells (microglia and macrophages). We recently showed that myeloid HDAC3 deletion promotes tissue repair and functional recovery after retinal ischemia-reperfusion (IR) injury via efferocytosis, a process by which myeloid cells engulf and clear apoptotic cells. Here, we investigated the mechanism by which myeloid HDAC3 deletion enhances efferocytosis. Employing an in vitro efferocytosis assay coupled with RNA sequencing on HDAC3 KO macrophages revealed that the secreted protein, CD5 molecule-like (CD5L), was the most upregulated among other pro-efferocytic genes. In vivo, we found that CD5L levels markedly increased in the retinas of myeloid HDAC3 KO mice subjected to IR injury, and its expression colocalized with myeloid cells. Co-immunoprecipitation experiments showed that HDAC3 represses CD5L expression in a liver X receptor (LXRα)-dependent manner. Additionally, we found that CD36, a receptor for CD5L that facilitates the clearance of apoptotic cells, was upregulated in retinal myeloid cells after IR. In vitro, CD5L treatment enhanced efferocytosis via CD36. We then evaluated the role of CD5L in retinal IR injury using in vivo neuronal, vascular, structural, and functional endpoints. CD5L KO mice showed worsened outcomes after IR, whereas treatment with recombinant CD5L was protective against retinal ischemic injury. Collectively, our findings suggest that deleting HDAC3 enhances macrophage efferocytosis by upregulating the CD5L/CD36 axis. CD5L may serve as a promising therapeutic target to improve outcomes in ischemic retinopathy.

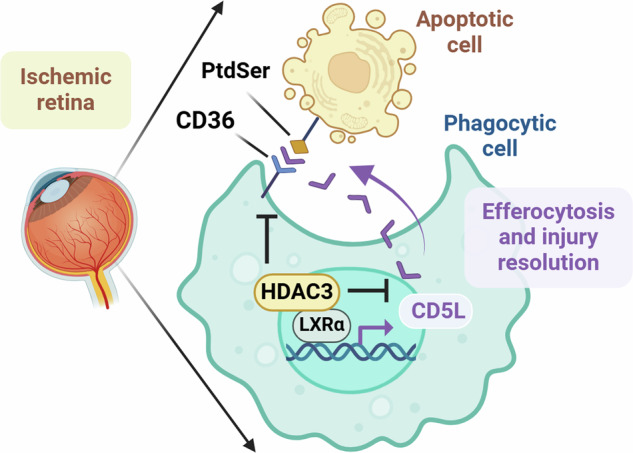

## Introduction

Ischemic retinopathies, such as diabetic retinopathy (DR), retinopathy of prematurity (ROP), and retinal artery and vein occlusions, are leading causes of visual impairment worldwide. These conditions impose a substantial financial strain and healthcare burden [1], yet current treatments offer limited efficacy [2]. Therefore, there is an urgent need to develop improved therapeutics. While the primary causes of ischemic retinopathies are diverse, they share a critical pathogenic event: an aberrant myeloid cell response to retinal injury that drives inflammation and tissue damage [3]. This myeloid cell response is widely studied using the mouse retinal ischemia-reperfusion (IR) injury model [4].

Efferocytosis is the process by which myeloid cells (e.g., macrophages/microglia) engulf apoptotic cells and damage-associated molecular patterns (DAMPs). It prevents the accumulation of apoptotic cells that can lead to a feed-forward cycle of inflammation and secondary necrosis [5, 6]. Efferocytosis is recognized as a reparative process in several pathological conditions, including stroke [7–9], cancer [10, 11], and atherosclerosis [5, 6, 12]. We recently demonstrated that efferocytosis is critical in resolving inflammation and promoting tissue repair following retinal IR injury. Critically, we identified the enzyme histone deacetylase 3 (HDAC3), a central mediator of the myeloid cell inflammatory response, as an intrinsic suppressor of efferocytosis and repair after retinal IR [3]. However, the specific molecular mechanisms by which HDAC3 inhibits myeloid cell-mediated efferocytosis and thus promotes retinal damage remain unknown.

To identify this mechanism of suppression, we focused on CD5 Molecule Like (CD5L), also known as Apoptosis Inhibitor of Macrophage (AIM). It is a soluble glycoprotein secreted mainly by macrophages in response to inflammation. It is known to enhance efferocytosis by acting as a ‘bridge’ molecule, binding to apoptotic cells and DAMPs to facilitate their subsequent engulfment by phagocytes [13, 14]. Reports have documented the protective role of CD5L in promoting efferocytosis after acute kidney injury and ischemic stroke [15, 16]. In the eye, CD5L is expressed by myeloid cells and retinal pigment epithelium (RPE) [17, 18]. It has been linked to increasing the phagocytic capacity of trabecular meshwork cells, thereby lowering the resistance to aqueous humor outflow [17]. Additionally, auto-antibodies against CD5L have been implicated in age-related macular degeneration, suggesting a protective role of CD5L in clearing abnormal lipid and protein depositions [17, 18]. These findings suggest a general protective role in clearing abnormal protein/lipid deposits and maintaining ocular health. Despite this, the function and regulation of macrophage-derived CD5L in promoting efferocytosis and mitigating neurovascular degeneration in the ischemic retina are entirely unknown.

Here, we identify a novel regulatory axis that governs the reparative phase after retinal ischemia. We report that HDAC3 deletion promotes CD5L-mediated efferocytosis by enhancing Liver-X-Receptor (LXR)-mediated CD5L transcription in retinal myeloid cells following IR injury. The resulting increase in CD5L signaling, mediated through the Cluster of Differentiation 36 (CD36) receptor on myeloid cells, facilitates the clearance of apoptotic cells, promotes injury resolution, and preserves retinal function. Furthermore, we demonstrate that therapeutic administration of recombinant CD5L confers significant protection against inner retinal neurovascular degeneration and preserves retinal function after IR injury.

## Results

### Macrophages lacking HDAC3 upregulate CD5L and other efferocytosis-related genes

**Fig. 1:**
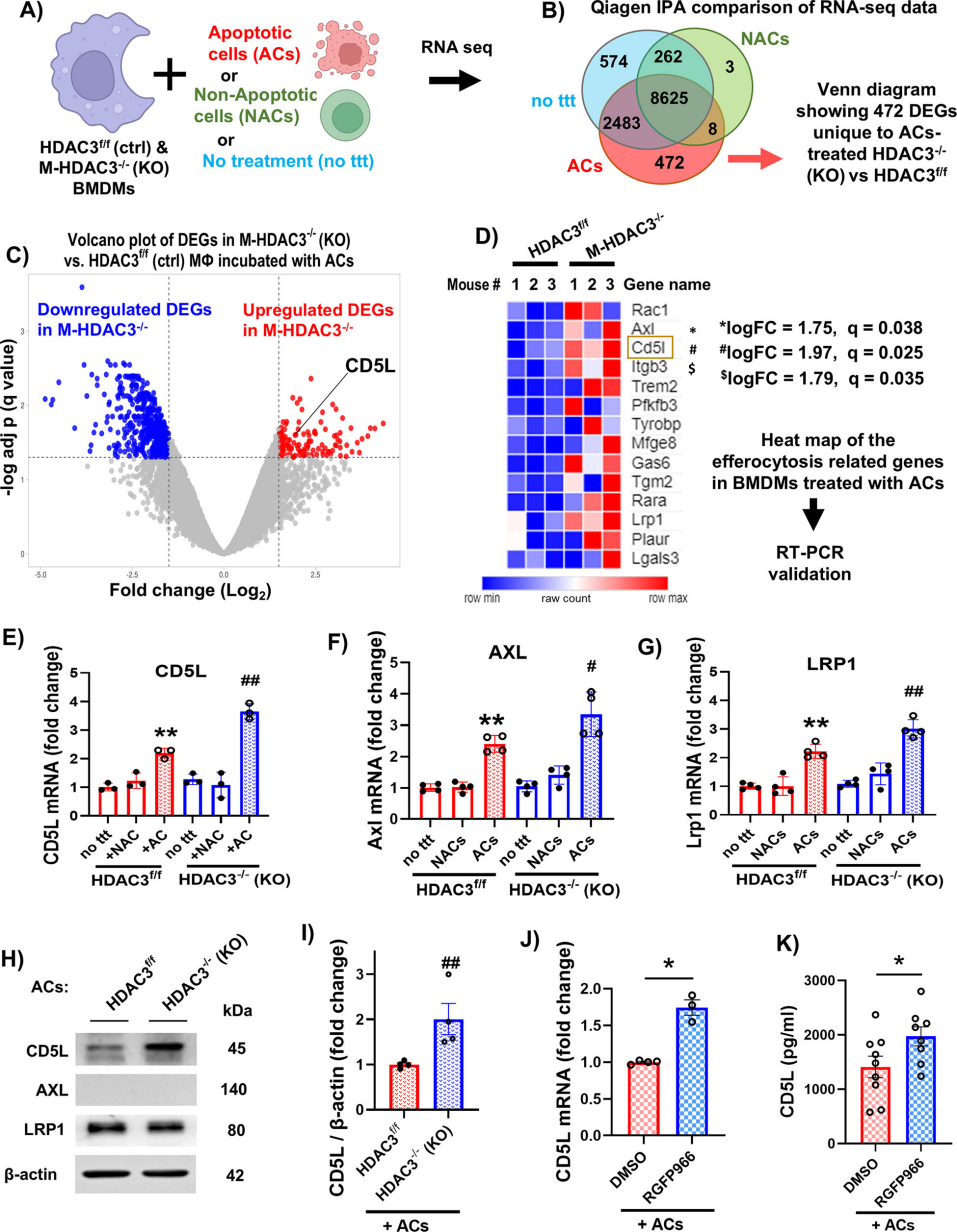
Macrophages lacking HDAC3 upregulate CD5L and other efferocytosis-related genes in vitro. **A** Schematic of the in vitro efferocytosis experiment using bone marrow-derived macrophages (BMDMs) isolated from M-HDAC3^−/−^ and HDAC3^f/f^ mice. RNA-seq and Qiagen Ingenuity Pathway Analysis (IPA) were performed on BMDMs subjected to no treatment (no ttt) or incubation with apoptotic cells (ACs) or non-apoptotic cells (NACs) for 45 minutes, followed by washing of unengulfed cells & incubation for 5 h, *n* = 3 biological replicates per group. **B** Venn diagram showing 472 differentially expressed genes (DEG) in RNA-seq data from ACs-treated HDAC3^−/−^ vs HDAC3^f/f^ BMDMs. **C** Volcano plot showing CD5L as one of the up-regulated DEGs in AC-treated HDAC3^−/−^ vs HDAC3^f/f^ BMDMs using log2 fold change (logFC) and *q* value (adjusted *p*) cutoffs of 1.5 and 0.05, respectively. **D** Heatmap displaying the upregulated efferocytosis-related genes in HDAC3^−/−^ BMDMs incubated with ACs. CD5L showed the highest logFC-value of 1.97 among the efferocytosis-related genes and a *q* value of 0.025. **E**–**G** RT-PCR validation of the efferocytosis-related genes CD5L, AXL, and LRP1 confirmed the RNA-Seq results showing upregulation of these genes in BMDMs derived from HDAC3^−/−^ vs HDAC3^f/f^ after incubation with ACs, *n* = 3–4, **P < 0.01 vs no ttt & NAC groups; ^#^*P* < 0.05, ^##^*P* < 0.01 vs all other groups. **H**, **I** Western blot and quantification of CD5L protein abundance confirm the RNA-seq finding that HDAC3 deletion upregulates CD5L expression in macrophages incubated with ACs, *n* = 4, ^##^*p* < 0.01 vs HDAC3^f/f^ groups. LRP1 protein levels did not differ between groups, whereas AXL was undetectable. **J** RT-PCR mRNA analysis of WT BMDMs treated with the HDAC3 inhibitor RGF966 shows upregulation of CD5L after incubation with ACs compared to DMSO-treated BMDMs, *n* = 3–4, *p < 0.05 vs DMSO. **K** ELISA confirms increased CD5L abundance in media from RGFP966-treated compared to DMSO-treated BMDMs after incubation with ACs, *n* = 8–9, *p < 0.05 vs DMSO.

We previously demonstrated that the enzyme HDAC3, a key regulator of the inflammatory response in myeloid cells, inhibits efferocytosis and tissue repair in the ischemic retina [3]. Here, we employed myeloid cell-specific HDAC3 KO (M-HDAC3^−/−^) mice to identify the mechanistic pathway by which myeloid HDAC3 deletion enhances efferocytosis and injury resolution after retinal ischemia. We identified HDAC3-regulated candidate genes using an in vitro efferocytosis assay in which bone marrow-derived macrophages were isolated from M-HDAC3^−/−^ and HDAC3^f/f^ mice and then incubated with either apoptotic cells (ACs), non-apoptotic cells (NACs), or no cells (untreated) before being subjected to RNA-seq (Fig. 1A, Supplementary Fig. 1A, B). We identified 472 differentially expressed genes (DEGs, adjusted *p* or *q* < 0.05) in ACs-treated HDAC3^−/−^ vs HDAC3^f/f^ macrophages (Fig. 1B, C). We then used Qiagen Ingenuity Pathway Analysis (IPA) to identify 14 genes that were related to efferocytosis and upregulated in the ACs-treated HDAC3^−/−^ macrophages (Fig. 1D). Of these 14 DEGs, the secreted protein CD5L was the most highly upregulated with logFC = 1.97 and *q* value = 0.025. Subsequently, RT-PCR results validated our RNA-seq data by confirming the upregulation of three DEGs with well-documented roles in efferocytosis, including CD5L, the pro-efferocytic receptors, AXL (a receptor tyrosine kinase), and the low-density lipoprotein receptor-related protein 1, LRP1 (Fig. 1E–G). We further validated the upregulation of CD5L in HDAC3^−/−^ macrophages treated with ACs by western blotting, whereas LRP1 did not show a change, and AXL was undetectable (Fig. 1H, I). Since HDAC3 can regulate gene expression independently of its histone deacetylase activity, we sought to determine whether HDAC3 enzymatic activity is required to suppress CD5L expression. We assessed CD5L mRNA in cell lysates and protein levels in the supernatant of macrophages treated with ACs with or without the HDAC3-specific inhibitor RGFP966. Indeed, we found a strong upregulation of CD5L mRNA (Fig. 1J) and secreted protein levels measured by ELISA (Fig. 1K) after pharmacological inhibition of HDAC3, suggesting that HDAC3 activity suppresses CD5L transcription induced by ACs treatment.

### CD5L is upregulated in HDAC3^−/−^ myeloid cells after retinal ischemic injury in vivo

**Fig. 2:**
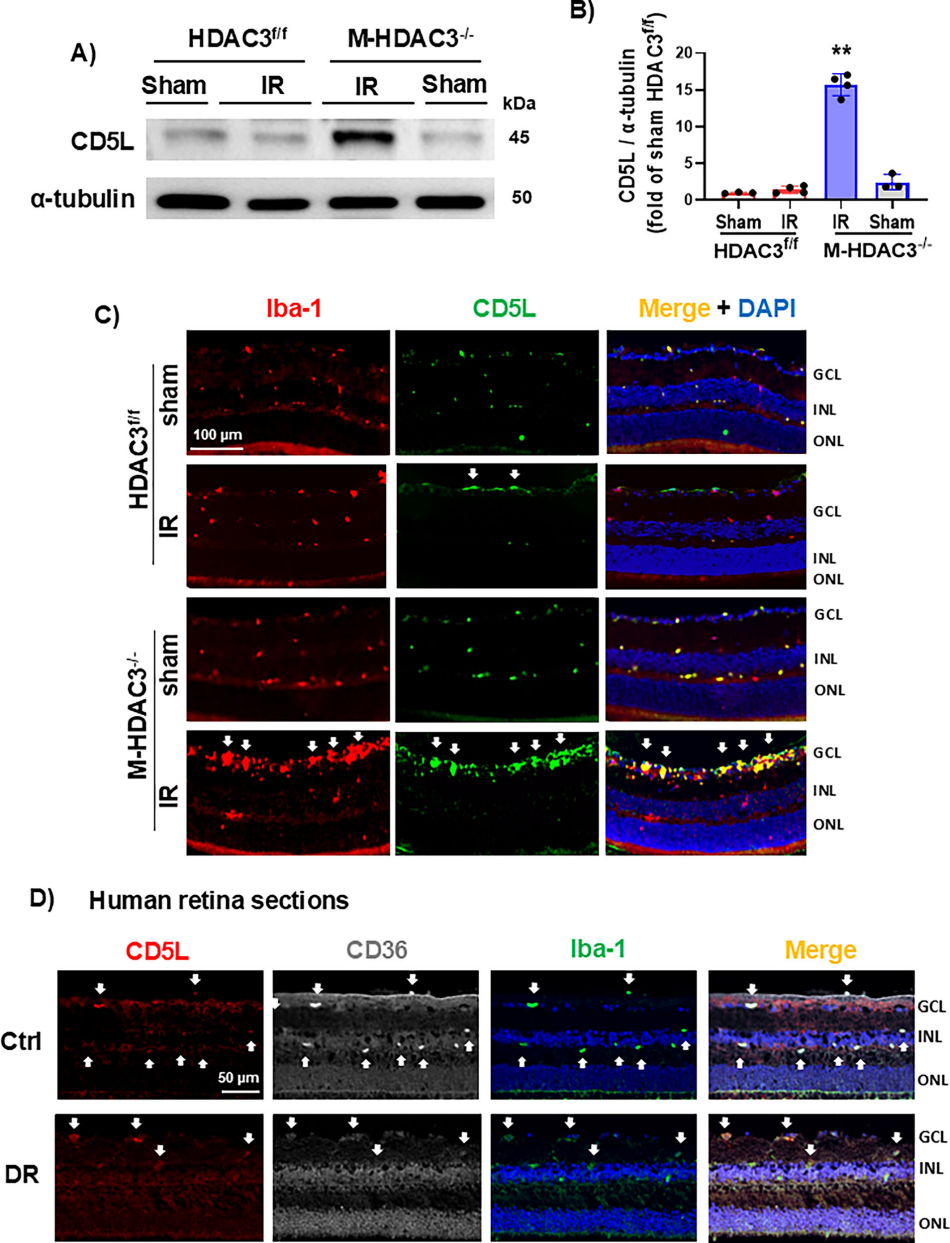
Myeloid HDAC3 deletion upregulates CD5L in retinas of IR-injured mice. **A**, **B** Western blot and quantification show upregulation of CD5L protein in the M-HDAC3^−/−^ retinas at 48 h after IR as compared to the HDAC3^f/f^ IR-injured retinas and sham controls, *n* = 3–4, **p < 0.01 vs other groups. **C** Representative images from retina sections immunolabeled for CD5L at 48 h after IR confirmed the upregulation of CD5L with strong colocalization with Iba-1^+^ microglia/macrophages (marked by arrows) in the M-HDAC3^−/−^ IR retinas, *n* = 2–4. **D** Postmortem retina sections from human donors show reduced colocalization (marked by arrows) of CD5L (red), and its receptor CD36 (white) in Iba-1^+^ cells (green) in DR retinas compared to healthy controls (Ctrl), *n* = 4.

After identifying CD5L as a pro-efferocytic target negatively regulated by HDAC3 in macrophages, we investigated its expression in vivo after retinal IR. CD5L was strongly upregulated in M-HDAC3^−/−^ retinas as compared to HDAC3^f/f^ retinas at 48 h after IR injury, implying that HDAC3 suppresses CD5L expression in the ischemic retina (Fig. 2A, B). We then determined that this over-abundant CD5L protein in the IR-injured HDAC3^−/−^ retinas strongly colocalized with Iba-1-labeled microglia/macrophages (Fig. 2C). This colocalization was most prominent in the inner retina, which is most adversely affected by IR. We also determined CD5L expression in postmortem human retina sections from control subjects and patients with DR, which involves retinal ischemia as a pathological component. Immunolabeling revealed strong colocalization of CD5L with its receptor, cluster of differentiation 36 (CD36), as well as with Iba-1-labeled myeloid cells in control retinas with a marked reduction in DR (Fig. 2D).

### HDAC3-LXRα association suppresses CD5L

**Fig. 3:**
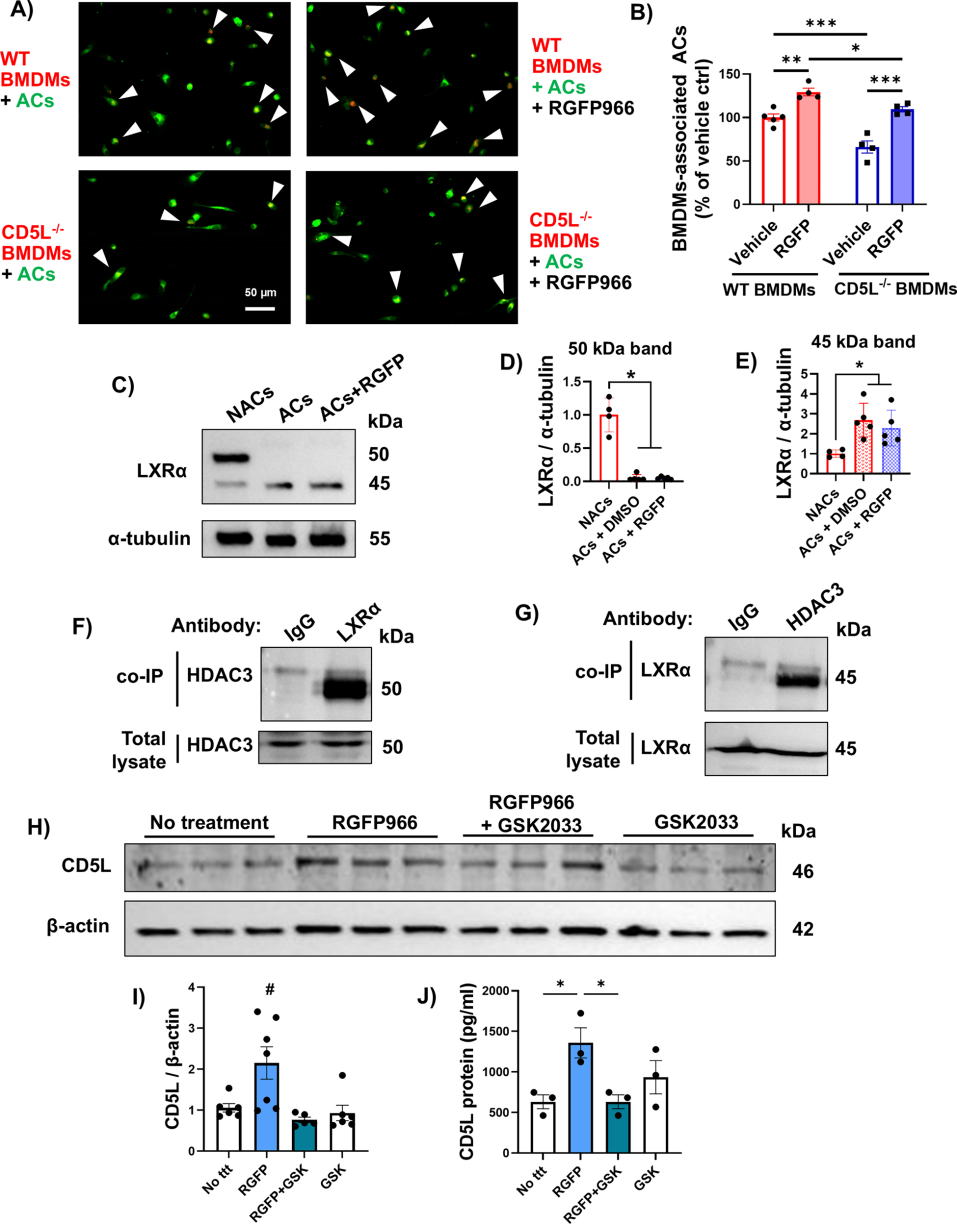
HDAC3 inhibition upregulates CD5L in an LXRα-dependent manner, promoting efferocytosis. **A** Representative images of in vitro efferocytosis studies in which CFDA green-labeled K-562 ACs were added to WT or CD5L^−/−^ (KO) CM-DiI red-labeled BMDMs pretreated with or without the HDAC3 inhibitor RGFP966 (arrows pointing to engulfed ACs). **B** Quantification shows enhanced efferocytosis with HDAC3 inhibition, whereas CD5L deletion reduced efferocytosis. Two-way ANOVA revealed that HDAC3 inhibition and CD5L deletion exerted statistically significant effects independently, with no interaction between the two factors, *n* = 4, *p < 0.05, **p < 0.01, ***p < 0.001. **C**–**E** Western blotting and quantification were performed on WT BMDMs incubated with NACs or ACs, showing changes in LXR-α, a transcription factor that regulates CD5L expression. Incubation with ACs with or without RGFP966 treatment led to reduced expression of the 50 kDa band and increased 45 kDa band as compared to incubation with NACs, *n* = 4–5, *p < 0.05. **F**, **G** Co-immunoprecipitation (co-IP) studies of WT BMDMs incubated with ACs suggest protein-protein interaction of HDAC3 and LXR-α, since treating cell lysates with antibodies for either protein resulted in detection of the other protein, but not with the IgG non-targeting antibody. **H**, **I** Western blot analysis on WT BMDMs incubated with ACs shows increased CD5L expression with RGFP966 treatment, whereas co-treatment with the LXR-α inhibitor GSK2033 blunted this effect, *n* = 5–7, ^#^*p* < 0.05 vs other groups. **J** ELISA analysis of secreted CD5L in the culture media confirmed the cell lysates’ Western blot results, *n* = 3, *p < 0.05.

We have previously reported that inhibition of HDAC3 by RGFP966 (RGFP) enhances the efferocytosis function of macrophages [3]. Here, we investigated whether CD5L mediates this enhanced efferocytosis when HDAC3 is inhibited. We subjected BMDMs from WT and CD5L KO (CD5L^−/−^) mice (Supplementary Fig. 2) to an in vitro efferocytosis assay with or without HDAC3 inhibition (Fig. 3A). As we reported previously [3], RGFP966-treated WT macrophages showed enhanced efferocytosis function when incubated with ACs, an effect that was reduced in the CD5L^−/−^ macrophages (Fig. 3B, Supplementary Fig. 4).

Next, we conducted a Qiagen IPA BioProfiler analysis of the RNA-seq data to investigate potential intermediates by which HDAC3 suppresses CD5L expression, thereby impairing the efferocytotic response. We identified the CD5L receptor, CD36, and liver-X-receptor α (LXRα), which transcriptionally mediates the expression of CD5L (Supplementary Fig. 1C) [13,19]. Because HDAC3 is known to repress LXRs-mediated gene transcription [20], we investigated whether HDAC3 suppression of LXRα attenuates CD5L expression in macrophages. Western blotting of protein lysates from BMDMs subjected to ACs revealed a lower apparent molecular weight of LXRα, suggesting that alternative splicing or post-transcriptional modification of LXRα is associated with efferocytosis (Fig. 3C–E). However, treatment with the HDAC3 inhibitor RGFP966 did not affect LXRα protein levels or its molecular weight. In contrast, reciprocal co-immunoprecipitation (co-IP) using anti-LXRα and anti-HDAC3 revealed that HDAC3 directly binds to LXRα (Fig. 3F, G). Treatment of BMDMs with the LXRα inhibitor, GSK2033, reduced the RGFP966-mediated upregulation of CD5L, suggesting that HDAC3 suppresses LXRα-mediated CD5L expression by inhibitory complex formation (Fig. 3H–J).

### Macrophage CD36 binds CD5L to mediate efferocytosis

**Fig. 4:**
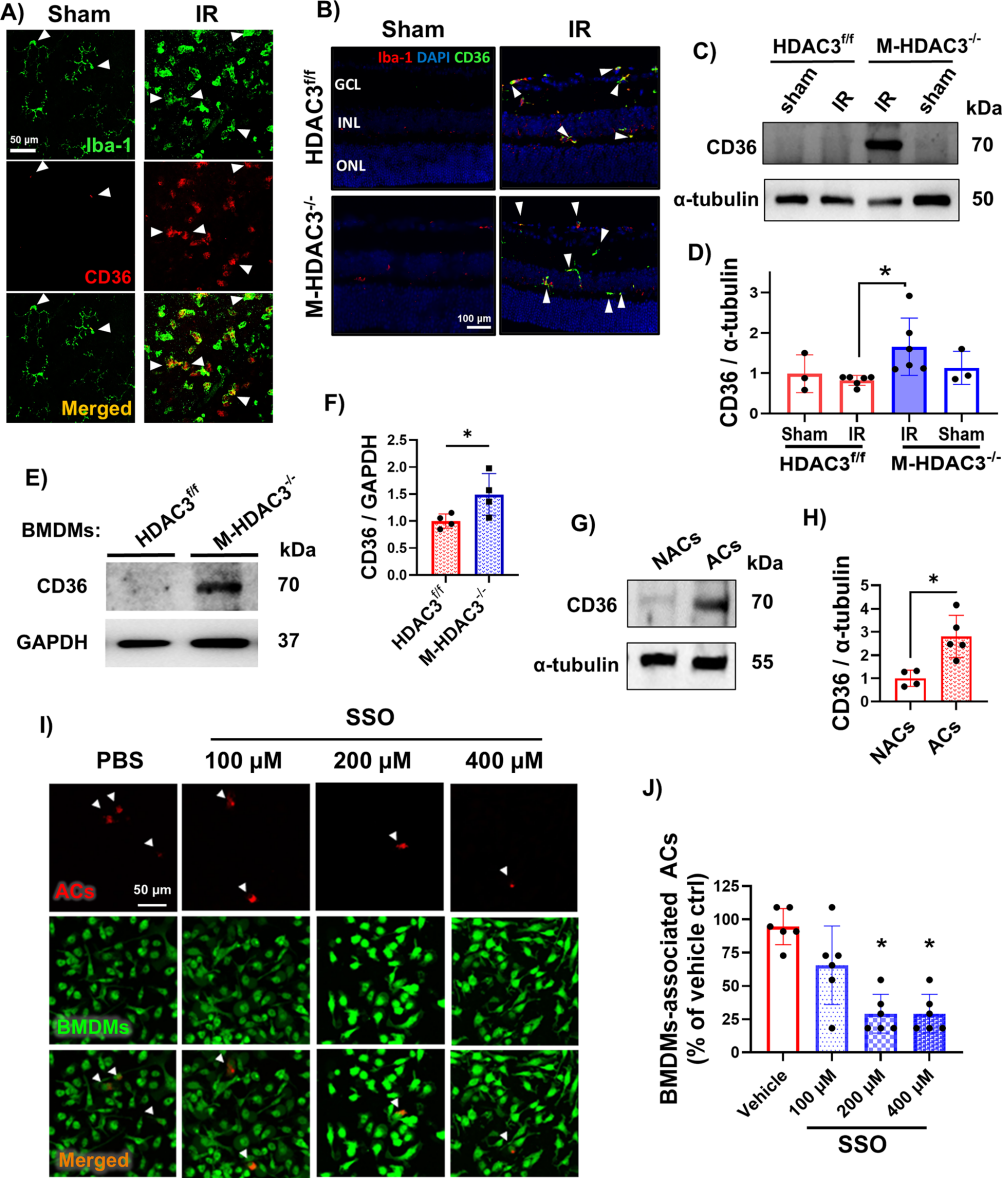
Myeloid HDAC3 deletion upregulates CD36 expression in vivo and in vitro. **A** Immunofluorescence labeling of CD36 (red) and Iba-1 (green) in WT sham and IR-injured retina flat mounts showed increased CD36/Iba-1 colocalization at 48 hours after IR as compared to sham. **B** Immunofluorescence labeling of retina sections with Iba-1 (red) and CD36 (green) shows increased CD36/Iba-1 colocalization in the injured retinas of M-HDAC3^−/−^ as compared to HDAC3^f/f^ mice at 48 h after IR, with less expression in sham retinas. **C**, **D** Western blot on retina lysates from M-HDAC3^−/−^ and HDAC3^f/f^ mice collected at 48 h after injury confirmed the increased CD36 protein in M-HDAC3^−/−^ retinas, *n* = 3-6, *p < 0.05. **E**, **F** Western blot on BMDMs lysates and quantification showed increased CD36 expression in HDAC3^−/−^ BMDMs as compared to HDAC3^f/f^ controls, *n* = 4, *p < 0.05. **G**, **H** Western blot on WT BMDMs cells lysates incubated with NACs or ACs showed elevated CD36 protein levels after ACs incubation, *n* = 4–5, *p < 0.05. **I** Representative images of an in vitro efferocytosis assay where CM-DiI red-labeled ACs were co-cultured with CFDA green-labeled WT BMDMs pretreated with various concentrations of the CD36 pharmacological inhibitor sulfo-N-succinimidyl oleate (SSO). **J** Quantification of BMDM-associated ACs as an indicator for efferocytosis showed decreased ACs uptake with increasing concentrations of SSO pretreatment, suggesting a key role of CD36 in macrophage efferocytosis ability, *n* = 6, *p < 0.05 vs vehicle and 100 µM SSO.

Our BioProfiler analysis of the RNA-seq data identified CD36, a multifunctional membrane glycoprotein expressed by many cell types, which acts as a receptor for CD5L. This finding implied that HDAC3 may not only suppress CD5L expression during retinal IR injury but also may suppress its target receptor to serially inhibit CD5L-mediated efferocytosis. We observed a strong colocalization of CD36 with Iba-1-labeled myeloid cells in the injured WT retina flat mounts collected at 48 h after IR (Fig. 4A). Immunolabeling of retina sections demonstrated more CD36-expressing myeloid cells in M-HDAC3^−/−^ IR retinas compared to HDAC3^f/f^ retinas (Fig. 4B), a finding confirmed by Western blots of retina lysates (Fig. 4C, D). Collectively, these data suggested that myeloid HDAC3 suppresses CD36 expression after retinal IR injury.

**Fig. 5:**
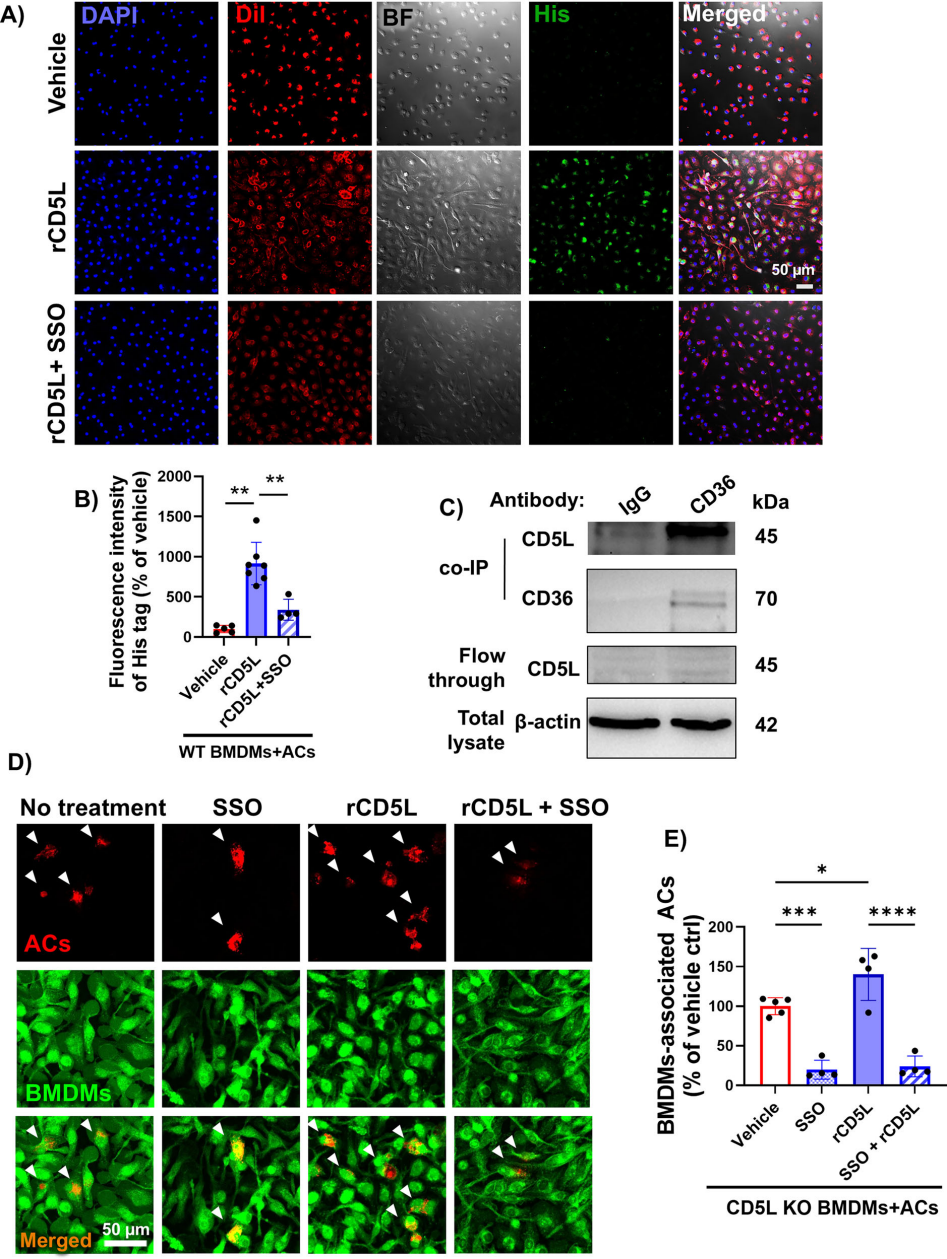
CD36 inhibition impairs CD5L-mediated efferocytosis. **A** Representative fluorescent and bright field (BF) images of CD5L uptake by BMDMs. WT CM-DiI red-labeled BMDMs were incubated with K-562 apoptotic cells (ACs) and treated with rCD5L (1 µg/ml) with or without sulfo-N-succinimidyl oleate (SSO, 200 µM) for 45 min, followed by washing. The His-tagged CD5L was labeled using anti-His secondary antibody (green) and counterstained with DAPI. **B** Quantification of rCD5L (anti-His-tag) fluorescence intensity in (**A**), which represents the internalized rCD5L during efferocytosis, shows reduced rCD5L uptake with the CD36 receptor blocker SSO, *n* = 4–7, **p < 0.01. **C** Co-immunoprecipitation (co-IP) using CD36 antibodies on WT BMDMs lysate after incubation with ACs and treated with CD5L suggests an interaction between CD36 and CD5L. **D** Representative images of WT CFDA green-BMDMs pretreated with or without SSO (200 µM) and incubated with CM-DiI red-labeled ACs in the presence or absence of rCD5L (1 µg/ml). **E** Quantification showed decreased CD5L-mediated efferocytosis with CD36 inhibition using SSO (200 µM), *n* = 4, *p < 0.05, **p < 0.01, ***p < 0.001, ***p < 0.0001.

In vitro studies confirmed that HDAC3 suppresses CD36 in macrophages and uncovered a crucial role for CD5L-CD36 signaling in efferocytosis. Initially, we conducted Western blots to confirm that HDAC3^−/−^ BMDMs exhibit higher CD36 expression compared to control HDAC3^f/f^ BMDMs (Fig. 4E, F). Next, we incubated WT macrophages with nonapoptotic (NACs) or apoptotic cells (ACs). We found that CD36 was strongly upregulated in response to ACs treatment, suggesting that an increased abundance of the receptor may enhance CD5L-mediated efferocytosis (Fig. 4G, H). Indeed, we observed that the CD36 inhibitor, SSO (200 and 400 µM), inhibited efferocytosis by WT BMDMs (Fig. 4I, J). Similarly, CD36-neutralizing antibodies also inhibited efferocytosis (Supplementary Fig. 3A, B). Finally, to verify that the macrophage CD5L/CD36 signaling pathway mediates efferocytosis, we repeated the in vitro efferocytosis assay using recombinant CD5L (rCD5L) and incubated a subset of WT BMDMs with SSO to block the CD36 receptor. Pharmacological blocking of the CD36 receptor with SSO inhibited CD5L binding to macrophages (Fig. 5A, B), and complementary co-IP studies showed that CD5L directly binds to CD36 during efferocytosis (Fig. 5C). Finally, the addition of rCD5L increased the uptake of ACs by WT BMDMs, whereas SSO-mediated inhibition of CD36 prevented this increased efferocytosis (Fig. 5D, E, Supplementary Fig. 4).

### CD5L deletion worsens retinal IR injury

**Fig. 6:**
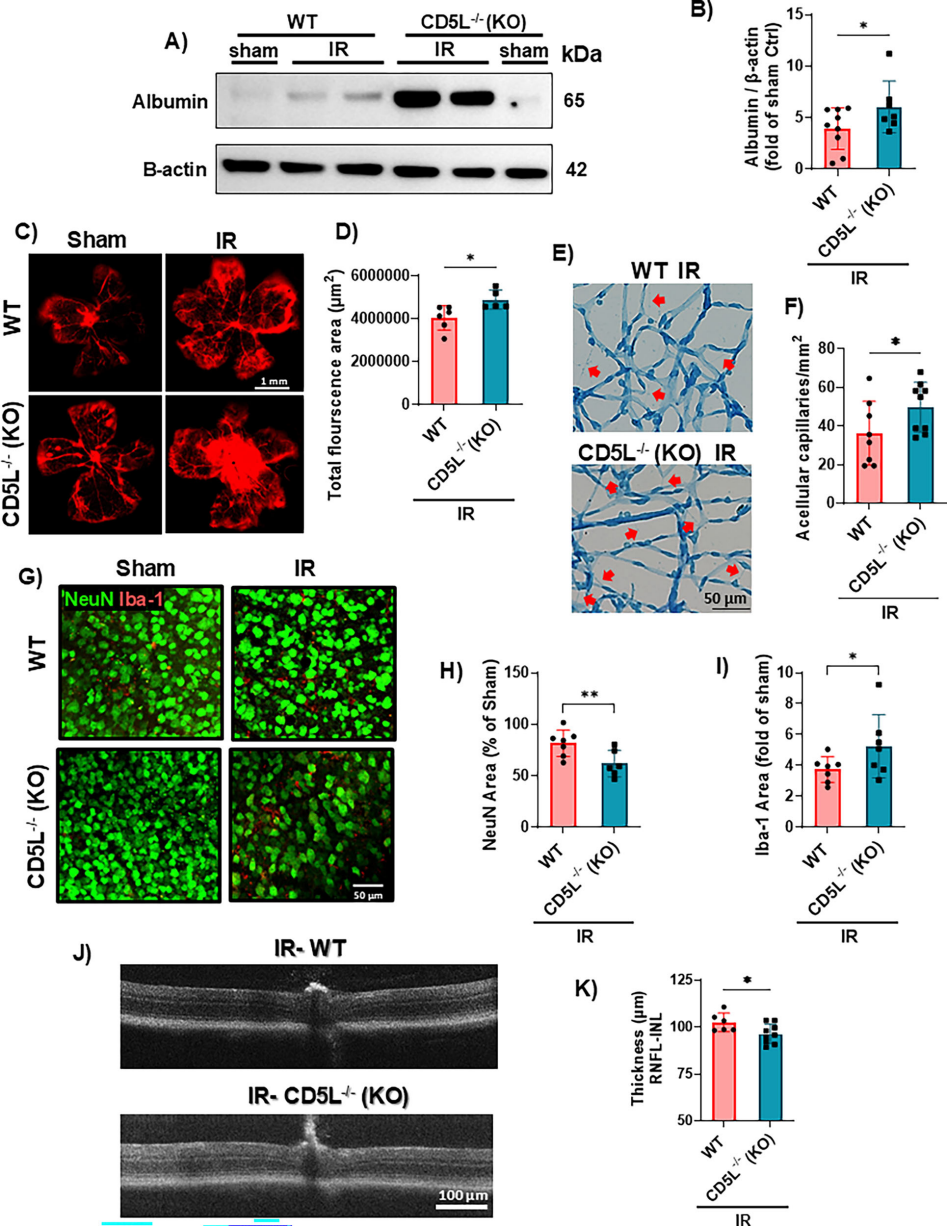
CD5L genetic deletion worsens IR-induced retinal vascular permeability, neurovascular degeneration, and retinal thinning. **A**, **B** Representative Western blot and quantification of retinal tissue albumin extravasation as a measure of permeability show increased albumin leakage in CD5L^−/−^ mice as compared to WT retinas 48 h after IR injury, *n* = 7–9, *p < 0.05. **C**, **D** Representative retina flat mount images of Evans blue leakage (red) and quantification of fluorescence area at 48 h post IR confirmed the albumin Western blot data, *n* = 5–6, *p < 0.05. **E**, **F** Representative images of trypsin-digested retinas and quantification of acellular capillaries (arrows) at 14 days after IR show increased acellular capillaries in injured CD5L^−/−^ retinas as compared to injured WT controls, *n* = 8–9, *p < 0.05. **G**–**I** Representatives of retina flat mounts immunolabeled with NeuN (green, neuronal marker) and Iba-1 (red, myeloid cell marker) and quantification show increased neuronal loss and Iba-1+ microglia/Macrophages in CD5L^−/−^ retinas at 7 days after IR, *n* = 7, *p < 0.05, **p < 0.01. **J**, **K** CD5L^−/−^ mice exhibited reduced inner retina thickness as measured by in vivo OCT at 14 days following IR injury, *n* = 6–9, *p < 0.05. Inner retina thickness was computed by InVivoVue software as the distance from the retinal nerve fiber layer (RNFL) to the inner nuclear layer (INL).

We next sought to determine the role of CD5L in retinal IR outcomes. CD5L^−/−^ mice subjected to sham or IR injury were sacrificed at different time points to assess neurovascular outcomes. The IR model results in blood-retinal barrier disruption at 48 h after injury, as assessed by Western blotting for blood albumin extravasation into the retinal tissue. CD5L^−/−^ retinas showed increased albumin leakage after IR compared to WT retinas (Fig. 6A, B), which was confirmed using flat mount imaging of leaked Evans blue dye (Fig. 6C, D), suggesting a vascular protective role of CD5L in IR. This vascular protective role was further confirmed by a retina trypsin digest 14 days after IR, which showed increased acellular capillaries in the CD5L^−/−^ retinas compared to WT retinas (Fig. 6E, F).

To assess neurodegeneration, we performed immunolabeling of flat-mounted retinas with the neuronal marker NeuN and the myeloid cell marker Iba-1 on day 7 after IR. Compared to WT IR retinas, CD5L^−/−^ retinas showed a decrease in the NeuN^+^ area and an increase in the Iba-1^+^ area, suggesting increased neurodegeneration and myeloid cell proliferation/infiltration after IR (Fig. 6G–I). The neurovascular degeneration detected in the CD5L^−/−^ mice after IR translated into decreased inner retinal thickness (Fig. 6J, K), suggesting an overall neurovascular protective role of CD5L and deleterious outcomes after *CD5L* gene deletion.

### Treatment with recombinant CD5L is neurovascular protective against retinal ischemic injury

**Fig. 7:**
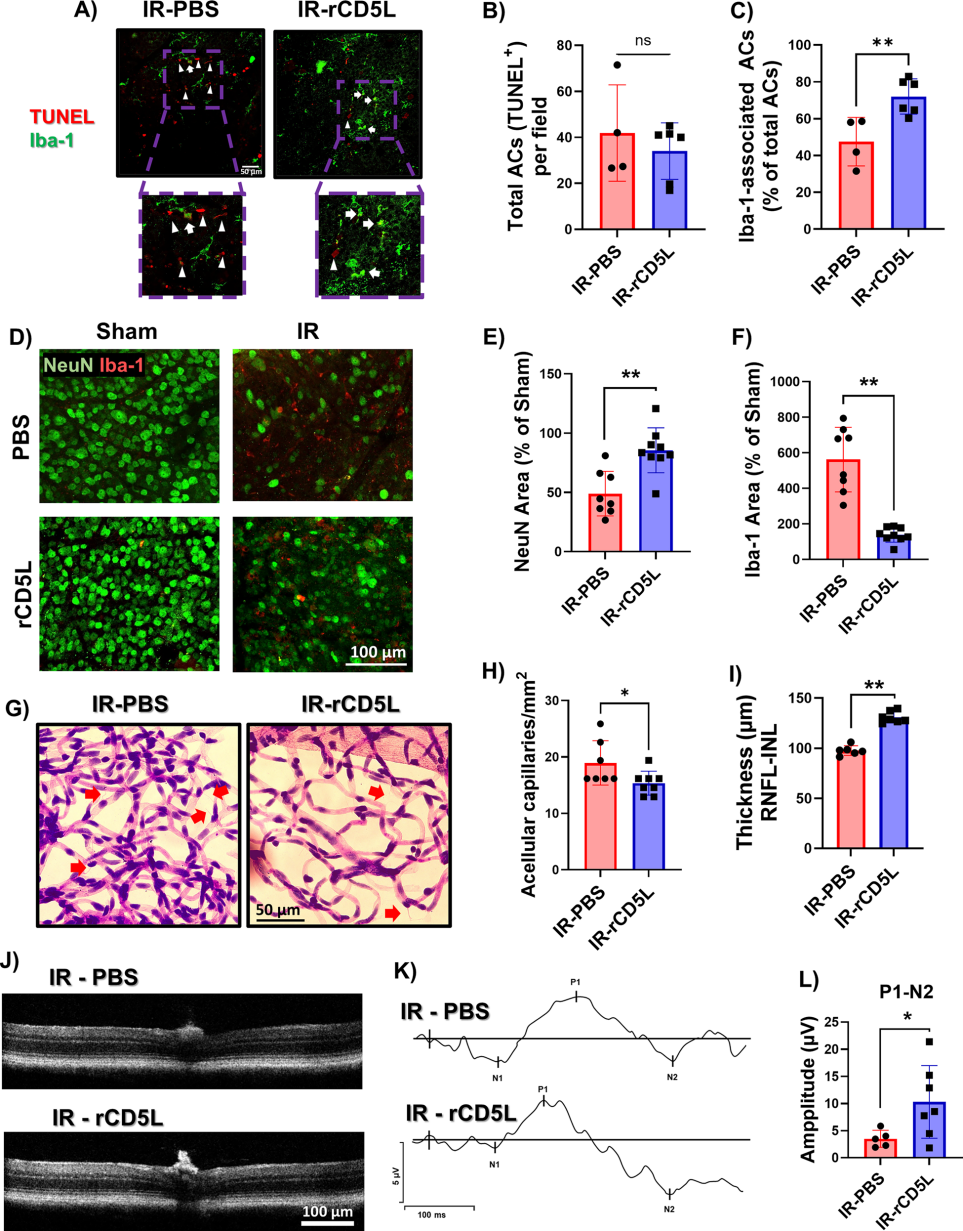
Treatment with recombinant CD5L promotes neuroprotection, mitigates retinal thinning, and preserves retinal function after IR injury. IR-injured WT mice received an intravitreal injection of rCD5L (1 μg/eye) or PBS (vehicle control) at 3 h post-injury and were then sacrificed at various time points. **A** Representative confocal images of ACs (TUNEL, red) and myeloid cells (Iba-1, green) immunolabeling of retinal flat mounts at 48 h after IR injury. **B** Quantification of total ACs shows no difference between the two groups, ns: not significant, *n* = 4–6. **C** Quantification of efferocytosis (% myeloid cell-associated ACs) shows improved efferocytosis in vivo (engulfment of ACs denoted by Iba-1/TUNEL colocalization) with rCD5L treatment. Arrows and arrowheads point to myeloid cell-associated and free TUNEL+ cells, respectively, *n* = 4–6. **p < 0.01. **D** Representative confocal images of immunofluorescent labeled retina flat mounts using NeuN to mark neurons (green) and Iba-1 (red) to mark microglia/macrophages at 7 days post-IR. **E**, **F** Quantification of neurons and microglia/macrophages area shows that rCD5L treatment protects injured retinas from neuron loss and reduces myeloid cell proliferation/infiltration as compared to control treatment, *n* = 8-9. **p < 0.01. **G**, **H** Vascular digests at 14 days after IR injury show a reduced number of acellular capillaries (red arrows) in injured WT retinas after rCD5L treatment as compared to vehicle-treated injured retinas, *n* = 7-8, *p < 0.05. **I**, **J** Representative OCT images and quantification show preserved inner retinal thickness with rCD5L treatment as compared to vehicle treatment at 14 days post-IR, *n* = 6–7,**p < 0.05. **K** Representative waveforms of pattern electroretinography (PERG) at day 7 after IR. **L** Quantification of amplitudes shows improved P1-N2 wave response in injured retinas treated with rCD5L as compared to vehicle treatment, *n* = 5–7,*p < 0.05.

To assess the pro-efferocytic response of the CD5L signaling pathway in vivo, we treated WT mice with recombinant CD5L (rCD5L) after IR and conducted an efferocytosis assay, labeling retina flat mounts collected at 48 h with Iba-1 and TUNEL to mark apoptotic cells. We found increased colocalization of Iba-1 with TUNEL+ cells in the retinas of rCD5L-treated mice, suggesting enhanced efferocytosis (Fig. 7A–C). We then assessed the neuroprotective effect of rCD5L treatment using retina flat mount immunolabeling at day 7 after IR. Treatment with rCD5L decreased neurodegeneration and myeloid cell proliferation/infiltration as assessed by NeuN and Iba-1 immunolabeling, respectively (Fig. 7D–F). However, the flow cytometric analysis at 2 days post-IR showed no differences in the % of microglia or myeloid leukocytes between injured retinas of WT mice treated with rCD5L or vehicle (Supplementary Fig. 5A–C). Notably, within the myeloid leukocyte population, rCD5L treatment significantly increased the frequency of Ly6CHigh monocytes, but not of Ly6C^intermed^ or Ly6C^low^ monocytes in the injured retinas (Supplementary Fig. 5D–G). Granulocytes and lymphocytes were not different between the injured groups (Supplementary Fig. 5H, I). We also assessed vascular degeneration using retina trypsin digests 14 days after IR, which showed a decrease in the number of acellular capillaries with rCD5L treatment. (Fig. 7G, H). Collectively, this neurovascular protective effect of CD5L treatment resulted in preserved retinal thickness and function after IR as measured by OCT (Fig. 7I, J) and ERG (Fig. 7K, L), respectively.

## Discussion

We recently discovered that efferocytosis is crucial for injury resolution in ischemic retinopathy and that efferocytosis is augmented in mice lacking myeloid HDAC3. Thus, myeloid HDAC3 deletion is neurovascular protective against retinal IR injury [3]. Here, we offer a mechanistic explanation for this observation and provide several novel findings. First, myeloid cells lacking HDAC3 exhibit an increased expression of the CD5L/CD36 pathway, which confers protection against retinal ischemic injury. Second, the increased expression of CD5L in HDAC3^−/−^ macrophages is mediated by disinhibition of LXRα-mediated transcription of CD5L. Third, CD5L exhibits neurovascular protective properties and facilitates efferocytosis by binding to CD36, thereby promoting the clearance of apoptotic cells. Fourth, the administration of recombinant CD5L mitigates the detrimental consequences of IR injury.

RNA sequencing and protein confirmation demonstrated that myeloid HDAC3 deletion or pharmacological inhibition strongly upregulates CD5L, suggesting that HDAC3’s enzymatic activity is involved in transcriptional repression of CD5L. Furthermore, HDAC3 suppression not only upregulates CD5L but also increases the expression of its receptor, CD36, providing a two-pronged mechanism to enhance efferocytosis capacity. The previously reported pro-efferocytic effect of HDAC3 inhibition was attenuated in CD5L^−/−^ macrophages, suggesting that CD5L plays a role in the enhanced efferocytosis observed with HDAC3 inhibition or deletion. However, statistical analysis using a two-way ANOVA revealed no interaction between HDAC3 inhibition and CD5L deletion, suggesting that other mediators, in addition to CD5L, contribute to this enhanced efferocytosis, and that HDAC3 inhibits efferocytosis via multiple mechanisms.

Earlier studies report that CD5L exerts a pro-efferocytic effect in models of acute kidney injury and stroke by acting as a bridging molecule between phagocytes and apoptotic cells [15,16]. As a bridging molecule, CD5L binds to phosphatidyl serine (PtdSer) on apoptotic cells to facilitate their recognition by macrophages. Here, we used CD5L^−/−^ mice as a genetic loss-of-function model to demonstrate the novel protective role of CD5L in ischemic retinopathy. CD5L^−/−^ mice exhibited significantly worse outcomes after IR injury, showing increased blood-retina barrier disruption, acellular capillaries, neurodegeneration, and overall inner retinal thinning. We also evaluated recombinant CD5L as a potential treatment to promote protective efferocytosis and preserve retinal structure and function. Treatment with recombinant CD5L in WT mice enhanced efferocytosis and provided robust neurovascular protection, leading to preserved retinal function and structure. Both our genetic and pharmacological studies demonstrated a protective role for CD5L in preserving retinal neurons, vasculature, and inner retinal thickness and function.

Although CD5L has multiple binding partners on different cell types, CD36 is suggested to be the main cell-surface receptor for CD5L on myeloid cells [21–23]. CD36 is a surface scavenger receptor found on myeloid cells and other cell types. It has drawn attention for its ability to bind and internalize long-chain fatty acids and oxidized low-density lipoprotein (ox-LDL), while also facilitating the clearance of apoptotic cells [24]. In the eye, CD36 reportedly aids in the clearance of ox-LDL and the age-related subretinal deposits associated with it [25]. Additionally, inhibition of CD36 has been reported to exacerbate photoreceptor degeneration in the outer retina [26]. In this study, we observed upregulation of CD36 on myeloid cells in M-HDAC3^−/−^ retinas subjected to IR injury and presented evidence that CD5L mediates apoptotic cell clearance by facilitating their binding to the CD36 receptor on macrophages.

Multiple reports indicate that the expression levels of CD5L and CD36 are increased by the nuclear receptor LXRα [19,27–31]. Additionally, other studies have shown that HDAC3 forms an inhibitory complex that disrupts LXRα-mediated gene transcription [32]. In our study, we used co-immunoprecipitation to directly demonstrate that HDAC3 binds to LXRα during efferocytosis, and inhibition of LXRα prevented the upregulation of CD5L in HDAC3^−/−^ macrophages. Thus, we provide evidence that HDAC3 inhibits LXRα-mediated transcription of CD5L, thereby mitigating the pro-efferocytotic action of CD5L that confers retinal neurovascular protection against IR injury.

Some limitations of our study should be acknowledged. First, we utilized global CD5L^−/−^ mice to investigate the CD5L/CD36 signaling pathway in macrophages, as conditional myeloid-specific CD5L^−/−^ mice are not readily available. Although CD5L is expressed mainly by myeloid cells, we cannot rule out the contribution of CD5L from non-myeloid cells [13]. For example, earlier reports indicate that RPE, in addition to myeloid cells, expresses CD5L [17, 18]. Although it seems unlikely that CD5L deletion from RPE would alter the course of inner retinal injury, we cannot ignore this possibility. A second limitation of our study is its focus on pro-efferocytic CD5L/CD36 signaling in macrophages alone, rather than in microglia, as obtaining retina microglia cultures in good yield is difficult. Flow cytometry showed that CD5L treatment has no effect on myeloid cell proliferation/infiltration after IR. The elevated presence of the pro-inflammatory Ly6C^High^ monocytes with CD5L treatment is surprising. However, Ly6C^High^ monocytes have also been linked to enhanced efferocytosis and cross-presentation [33]. Additional limitations relate to the in vitro efferocytosis assays since they were not conducted with retinal cells. Instead, we used human K562 lymphoblasts, as they are readily induced to undergo apoptosis and their suspension nature allows easy washing after incubation with macrophages. Furthermore, we acknowledge that differences in engulfment efficiency between HDAC3 KO and control macrophages may have skewed the RNA-seq results. To mitigate the risk of confounding apoptotic cell cargo mRNA, we used mouse BMDMs co-incubated with human K562 cells as the source of apoptotic cells. We then performed RNA-seq alignment exclusively using a mouse reference genome, thereby effectively minimizing signal from the ingested human K562 mRNA.

In conclusion, this study shows that myeloid cells lacking HDAC3 upregulate the CD5L/CD36 axis, a novel regulator of reparative efferocytosis in the ischemic retina. CD5L binds to CD36 to confer neurovascular protection by enhancing efferocytosis and promoting the clearance of apoptotic cells. The robust neurovascular protection afforded by recombinant CD5L treatment underscores its potential as a therapeutic agent to enhance injury resolution and improve visual outcomes in patients with ischemic retinopathies.

## Materials and methods

### Mice

All animal experimental procedures were approved by the Institutional Animal Care and Use Committee (IACUC) of the University of Arkansas for Medical Sciences and performed in accordance with the Association for Research in Vision and Ophthalmology (ARVO) guide for the care and use of laboratory animals. CD5L^−/−^ Knockout (KO) mice were backcrossed to the C57BL/6J background for at least 12 generations. Constitutive myeloid-specific HDAC3 KO (M-HDAC3^−/−^) mice were developed by crossing C57BL/6J floxed mice with LoxP sites on either side of exons 7 of HDAC3 (HDAC3^f/f^, originally developed by Dr. Scott W. Hiebert) to LysM^Cre^ mice (*Lyz2*^*Cre*^, Jackson lab stock # 004781) [34]. Wild-type (WT) C57BL/6J mice were obtained from the Jackson Laboratory (stock # 000664). We randomized animals to experimental groups by an independent technician.

### Mouse retinal ischemia-reperfusion (IR) model and treatments

Retinal IR injury was induced in mice (10–12 weeks old) as we previously described [3, 35–37]. Briefly, a 36-gauge needle connected to a raised saline bag was inserted into the anterior chamber of the right eye to induce IOP elevation in mice under ketamine/xylazine anesthesia. The elevated IOP was maintained for 60 min to induce ischemia, followed by needle removal to allow reperfusion. The left eye served as a sham control.

For CD5L treatment studies, WT mice received a single dose of 1 μg in 1 μL of recombinant CD5L (rCD5L, R&D Systems™, Cat. #2834CL050) or phosphate-buffered saline (PBS, vehicle control) administered intravitreally at 3 h post-IR.

### Human eye sections

Normal and DR postmortem human eye paraffin-embedded sections were obtained from the National Disease Research Interchange (NDRI, Philadelphia, PA). Sections were deparaffinized and rehydrated, and antigen retrieval was performed as we previously described [3]. Sections were permeabilized and blocked with 3% normal donkey serum (MilliporeSigma, Cat. # 5058837) and 3% bovine serum albumin (BSA) in 0.2% Triton X-100 for 1 h. Sections were then incubated with primary antibodies and fluorescent secondary antibodies, then cover-slipped with Vectashield antifade mounting medium containing 4′,6-diamidino-2-phenylindole (DAPI; Vector Laboratories, Newark, CA). Primary antibodies used were as follows: Iba-1 (FUJIFILM Wako, Cat. # 011-27991), CD5L (Abcam, Cat. #AB45408) and CD36 (GeneTex, Cat. # GTX42052).

### Immunofluorescence and image analysis

Immunofluorescence staining was conducted as we described previously [3, 35–37]. Briefly, eyeballs were fixed in 4% paraformaldehyde (PFA), then dissected into retina flat mounts or dehydrated/cryoprotected in 30% sucrose and embedded in optimal cutting temperature (O.C.T.) for cryosectioning. Retina flat mounts or cryostat sections were permeabilized in 0.1% Triton X-100 and blocked in 10% donkey serum and 1% BSA for 1 h. Primary antibodies were incubated overnight at 4 °C, followed by washing in PBS and incubation of secondary antibodies at room temperature for 1 h. Images were captured using a Zeiss LSM 880 Airyscan inverted confocal microscope, and image analysis was performed in ImageJ.

### TUNEL staining and in vivo efferocytosis assay

To quantify in vivo efferocytosis, a TUNEL assay was performed using Click-iT™ Plus TUNEL Assay (Invitrogen™) according to the manufacturer’s instructions on retina flat mounts at 48 h following IR, as we previously reported [3]. The TUNEL-stained flat mounts were further immunolabeled for the myeloid cell marker Iba-1 and imaged. The total number of TUNEL^+^ apoptotic cells (ACs), Iba-1^+^ macrophages, and TUNEL^+^Iba-1^+^ cells was then counted. Efferocytosis was calculated and presented as a percentage of ACs engulfed by macrophages/microglia ([number of Iba-1 ^+^ TUNEL^+^ cells ÷ total number of TUNEL+ cells] × 100).

### Electroretinography

The Celeris electroretinogram system (Diagnosys LLC, Cambridge, UK) was used to assess retinal PERG function. Measurements were conducted on IR-injured mice and sham controls as we previously described [3]. Briefly, the mice were dark-adapted overnight, then anesthetized with a ketamine/xylazine mixture, and their pupils were dilated with 0.5% tropicamide (Akron Pharmaceuticals, IL) and 1.25% phenylephrine. PERG was performed under dim red lighting. Eyes were assessed using a configuration where one pattern stimulator was used for the test eye, and a full-field stimulator served as a reference electrode for the contralateral eye. Retinal function was quantified using the amplitudes of the P1 and N2 peaks, and the difference between these components |P1-N2|.

### Optical coherence tomography (OCT)

OCT images were captured using an Ophthalmic Imaging System (Bioptigen, Durham, NC) as we previously described [3]. In brief, the eyes of mice were dilated with 1% tropicamide (Akron Pharmaceuticals, IL) and anesthetized with a ketamine/xylazine mixture. Mice were placed on a mouse holder to fix the animal’s posture for scanning. Retina rectangular scans (3 frames/scan, 1000 A scan/B scan × 100 B scan, 1.4 mm × 1.4 mm) were captured. The averages of the three OCT image scans, as determined by InVivoVue software (Bioptigen Inc., Durham, NC, USA), were used to measure the thickness of the retinal layers.

### Retinal vascular permeability assays

#### Evans blue leakage

Retinal vascular permeability in vivo was evaluated at 48 h after IR injury by imaging the extravasation of Evans Blue dye as previously described by us and others [3, 38]. Mice were anesthetized and given a transcardial injection of (200 µL, 2% in normal saline) Evans Blue in normal saline. After five minutes, the anesthetized mice were euthanized, and their eyes were fixed for 24 h in 4% PFA. Retinal flat mounts were prepared, mounted in Vectashield antifade medium (Vector Laboratories, Newark, CA, USA), and imaged using a fluorescence microscope. The total leakage area was quantified using ImageJ by thresholding the red (Evans Blue) channel, subtracting the background, binarizing the fluorescence area, and measuring the resulting binary area across the entire retina.

#### Albumin extravasation

The extravasation of albumin to the retina was measured as described by us and others [3, 39, 40]. Briefly, Injured mice were deeply anesthetized and transcardially perfused with cold PBS for 5 min to rinse out intravascular blood cells and proteins. Retinas were then collected and homogenized in Radioimmunoprecipitation Assay (RIPA) buffer, and albumin levels were assessed using Western blot to serve as a measure of vascular permeability and leakage.

### Retinal vasculature isolation and acellular capillaries quantification

We employed the trypsin digestion method to isolate and image the retinal vasculature, as previously described by us and others [3, 36, 39, 41]. In brief, on day 14 after IR injury, the eyes were enucleated and fixed in 4% PFA overnight. The retinas were then dissected and placed in a trypsin solution (#15090046, ThermoFisher Scientific, Waltham, MA) at 37 °C. Following trypsin digestion, the retinas were cleaned under a dissecting microscope, and the vasculature was mounted on silane-coated slides and air-dried. The retinal vasculature was stained with periodic acid-Schiff and hematoxylin, dehydrated, and covered with a coverslip. Images of random fields of the mid-retina were captured using a brightfield microscope. The number of acellular capillaries was manually quantified and calculated per 1 mm² of retina.

### Flow cytometry analysis of the immune cell populations in the retina

Cells from retinal tissue digests (2–4 retinas pooled per preparation) were isolated, filtered, and labeled for flow analysis as we previously described [3, 42]. Briefly, the cells were incubated with a viability dye (Fixable Viability Dye eFluor 450, eBioscience) for 30 min, then washed three times with cold PBS. Cells were resuspended in 5% BSA and blocked with 1 μg/mL of Fc receptor blocking anti-mouse CD16/32 antibody and 20% normal rat serum for 10 minutes at room temperature. Subsequently, cells were stained with labeled antibodies that included PerCP-Cy5.5-conjugated rat anti-mouse CD11b monoclonal antibody (1:100, Clone M1/70, BD Bioscience, Cat. #BDB550993), APC-Cy7-conjugated rat anti-mouse CD45 monoclonal antibody (1:100, Clone 30-F11, BD Bioscience, Cat. #BDB557659), PE-conjugated rat anti-mouse Ly6C monoclonal antibody (1:100, Clone HK1.4, eBioscience, Cat. #50-245-507), and FITC-conjugated rat anti-mouse Ly6G (Gr-1) monoclonal antibody (1:100, Clone RB6-8C5, eBioscience, Cat. #50-991-9) for 25 min on ice. The immunolabeled cells were washed three times with cold PBS and fixed with 0.4% PFA. Data were acquired using a BD LSRFortessa flow cytometer (BD Biosciences) and analyzed with FlowJo software (Tree Star Inc., San Carlos, CA, USA).

### Bone marrow-derived macrophage (BMDMs) culture

In vitro experiments were conducted using bone marrow-derived macrophages (BMDMs). Bone marrow was obtained from femurs and differentiated into macrophages in vitro as we previously described [3]. Briefly, bone marrow cells were isolated in cold PBS, filtered, and resuspended in a DMEM high-glucose medium containing 20% fetal bovine serum (FBS, Gibco; Thermo Fisher, NY) with 20% L929 cells’ conditioned media and 1% penicillin-streptomycin to induce macrophage differentiation. Cells were seeded in 100 mm dishes and allowed to adhere. After 4 days, the media was replaced with fresh differentiation media, and experiments were conducted on day 7.

### Culture of K562 cells and induction of apoptosis

The human K562 lymphoblast suspension cells were cultured in RPMI 1640 medium (Gibco; Thermo Fisher, NY) containing 2 mM L-glutamine supplemented with 10% FBS (Gibco; Thermo Fisher, NY) and 1% penicillin-streptomycin (Gibco; Thermo Fisher, NY), and maintained in a 5% CO_2_ incubator at 37 °C.

Apoptosis was induced in K562 cells as we previously described [3]. Briefly, the suspended K562 cells were subjected to UV-B irradiation for 15 min using a UV crosslinker (Spectrolinker XL-1500, Spectronics Corporation, Melville, NY). Cells were then resuspended in fresh DMEM media with 10% FBS (Gibco; Thermo Fisher, NY, USA) and incubated in a 5% CO_2_ incubator at 37 °C for two hours.

### In vitro efferocytosis and evaluation of the gene and protein expression in macrophages

We subjected BMDMs to efferocytosis assays using K562 cells to evaluate the gene and protein expression of efferocytosis-related markers in macrophages, as previously reported [3].

Depending on the experiment, BMDMs derived from WT or HDAC3^f/f^ and M-HDAC3^−/−^ mice were co-cultured with apoptotic K562 cells for 45 minutes at 37 °C. Non-apoptotic cells were used as controls. After incubation, the unengulfed suspended K562 cells were washed with PBS. The BMDMs were then maintained for 5 h for total RNA isolation, or 18 h for protein extraction and Western blotting. BMDMs were homogenized in TRIzol reagent (Invitrogen, CA, USA) for total RNA extraction or RIPA buffer for protein extraction. Culture media were collected for ELISA analysis. The quantity and integrity of RNA were evaluated using NanoDrop and TapeStation (Agilent Technologies, Palo Alto, CA, USA). When needed, BMDMs were pre-treated for 2 h with the LXRα inverse agonist GSK2033 (1 μM, Tocris, Cat. #5694) or the specific HDAC3 inhibitor RGFP966 (2 μM, Sigma, St. Louis, MO, USA) with DMSO as the solvent control.

### RNA sequencing and analysis

Total RNA was isolated from BMDMs derived from HDAC3^f/f^ and M-HDAC3^−/−^ mice (3 animals per group) and subjected to the efferocytosis assay in vitro using K562 cells. The RNA samples were sent to Azenta (Indianapolis, IN, USA) for sequencing. The RNA sequencing libraries were prepared using the NEBNext Ultra II RNA Library Prep Kit for Illumina using the manufacturer’s instructions (New England Biolabs, Ipswich, MA, USA).

The samples were sequenced using a 2×150 bp paired-end (PE) configuration. RNA-seq alignment was performed using a mouse reference genome, effectively minimizing signal from the ingested human K562 mRNA. After extracting the gene hit counts, the gene hit counts table was used for downstream differential expression analysis. Using DESeq2, gene expression was compared between the groups. The Wald test was used to generate *P* values and Log2 fold changes. Genes with adjusted *p*-values < 0.05 and absolute log2 fold changes >1 were considered as differentially expressed genes (DEGs) for each comparison. Heatmaps and clustering were done using the online BioJupies tool (https://maayanlab.cloud/biojupies). The data have been deposited in the Gene Expression Omnibus (GEO) repository at the National Center for Biotechnology Information (NCBI) and are accessible through accession number GSE301077.

Principal Component Analysis (PCA) was performed to visualize similarities in gene expression across samples in the RNA-seq dataset. The potential biological significance was analyzed using Ingenuity Pathway Analysis (IPA) BioProfiler (Qiagen Bioinformatics, Redwood City, CA, USA).

### Real-time PCR analysis of efferocytosis-related genes in BMDMs

Total RNA was reverse transcribed using the Verso cDNA Synthesis Kit (Fisher Scientific, NJ, USA). Real-time PCR was performed using PowerTrack SYBR Green Master Mix (Applied Biosystems) and a CFX96 Touch Real-Time PCR Detection System (Bio-Rad). The data were analyzed using the comparative Ct method with GAPDH as an internal control. The following primer sequences were used: CD5L forward, GAG GAC ACA TGG ATG GAA TGT; CD5L reverse, ACC CTT GTG TAG CAC CTC CA; Lrp1 forward, ACTATGGATGCCCCTAAAACTTG; Lrp1 reverse, GCAATCTCTTTCACCGTCACA; Axl forward, ATGGCCGACATTGCCAGTG; Axl reverse, CGGTAGTAATCCCCGTTGTAGA.

### ELISA analysis of secreted CD5L in BMDMs-derived media

Media CD5L quantification was performed using a Mouse CD5L ELISA kit (Invitrogen™) in accordance with kit instructions.

### Western blotting

Retinas or BMDM’ total proteins were extracted with RIPA lysis buffer, and protein concentrations were determined with a Bicinchoninic Acid (BCA) protein concentration assay kit (Pierce BCA Protein Assay Kit, Thermo Fisher Scientific). Western blotting was conducted as previously described [3]. The membranes were incubated overnight with primary antibody solutions (HDAC3, Cell Signaling, Cat. # 3949T/S; CD5L, Abcam, Cat. # AB45408; CD36, GeneTex, Cat. # GTX42052, albumin, Proteintech, Cat. # 16475-1-AP; AXL, R&D, Cat. # AF854-SP; LRP1, Cell Signaling, Cat. # 64099S; LXRα, Proteintech, Cat. # 14351-1-AP; β-actin, Sigma, Cat. # A5441; and tubulin, Sigma, Cat. # T9026) at 4 °C. The next day, HRP-conjugated secondary antibodies (Invitrogen) were diluted 1:2000 in 5% milk and incubated with the membranes at room temperature for 1 h.

### Evaluation of macrophages’ efferocytic capacity in vitro

Experiments were conducted using K562 cells to assess macrophage efferocytotic capacity in vitro. BMDM derived from WT mice and CD5L^−/−^ mice were labeled with Vybrant® CM-DiI cell-labeling solution (Molecular Probes, Cat. # V-22888), washed, and seeded on glass-bottom 35-mm dishes. The labeled BMDMs were treated with or without the HDAC3 inhibitor RGFP966 (2 μM) for 2 h. Then, BMDMs were co-cultured with apoptotic K562 cells (labeled with Vybrant® CFDA SE cell tracer Kit, Invitrogen, Carlsbad, CA) at a 1:1 ratio for 45 min at 37 °C, followed by washing of un-engulfed K562 suspension cells with PBS. The dishes were imaged using a confocal microscope, with random images taken from different areas of each dish. In separate experiments, macrophages were suspended and analyzed by flow cytometry to confirm the results obtained by confocal imaging.

When needed, BMDMs were pre-treated for 2 h with the CD36 inhibitor sulfo-N-succinimidyl oleate (SSO, 100-400 μM), a CD36 neutralizing antibody (1–4 μg/mL, GenTex, Cat. # GTX42052), or vehicle control. In other experiments, BMDMs were treated with rCD5L (1 μg/mL) tagged at the C-terminus with a 6-His tag to facilitate immunofluorescent analysis using an anti-6x-His primary antibody (Invitrogen, Cat. # PA1-983B).

### Co-immunoprecipitation (co-IP)

BMDMs were plated in a 12-well plate and subjected to the efferocytosis assay as described above. After washing the apoptotic K562 cells, the BMDMs were incubated for 18 h. Then, the media were collected, and co-IP assays were performed using Abcam’s Immunoprecipitation Kit (Abcam, Cat. # ab206996) according to the manufacturer’s instructions. In brief, cells were lysed in RIPA lysis buffer with protease inhibitor, followed by rotary mixing for 30 min at 4 °C. The lysates were then cleared by centrifugation at 10,000 × *g* for 10 min at 4 °C and immunoprecipitated with the following antibodies: HDAC3 (Invitrogen, Cat. # PA5-29026), LXRα (NR1H3, Proteintech, Cat. # 14351-1-AP), CD36 (GeneTex, Cat. # GTX42052) or IgG isotype control (GeneTex, Cat. # GTX35009) bound to Protein A/G Sepharose® beads for 2 h at 4 °C. Beads were cleared by centrifugation at 1000 × *g* for 3 min and the flow through was obtained. Then, the beads were washed three times with wash buffer, followed by centrifugation at 1000 × *g* for 2 min, with a 5-min rotation in between. Proteins were then eluted from the beads with 2× Laemmli loading buffer at 95 °C for 5 min. Co-immunoprecipitated proteins were analyzed by immunoblotting.

### Statistical analysis

Statistical significance between two or more groups was determined using Student’s *t* test or analysis of variance (ANOVA) with Tukey’s post hoc test, respectively. A *p*-value < 0.05 was considered statistically significant. Statistics and graphs were performed using GraphPad Prism 10 software, and data were presented as mean ± standard deviation (SD).

## Supplementary information

Original data

Supplementary data

## Data Availability

RNA sequencing data supporting the findings of this study have been deposited in GEO under accession GSE301077. For further requests or additional details, please contact the corresponding author.
