# Supplementary material for: CD5L promotes efferocytosis and resolution of retinal ischemic injury
Source: Cell Death Dis. 2026 Apr 20;17(1):520. doi: 10.1038/s41419-026-08752-8 (PMC13222871; doi:10.1038/s41419-026-08752-8)

Uncropped Western blots

F1

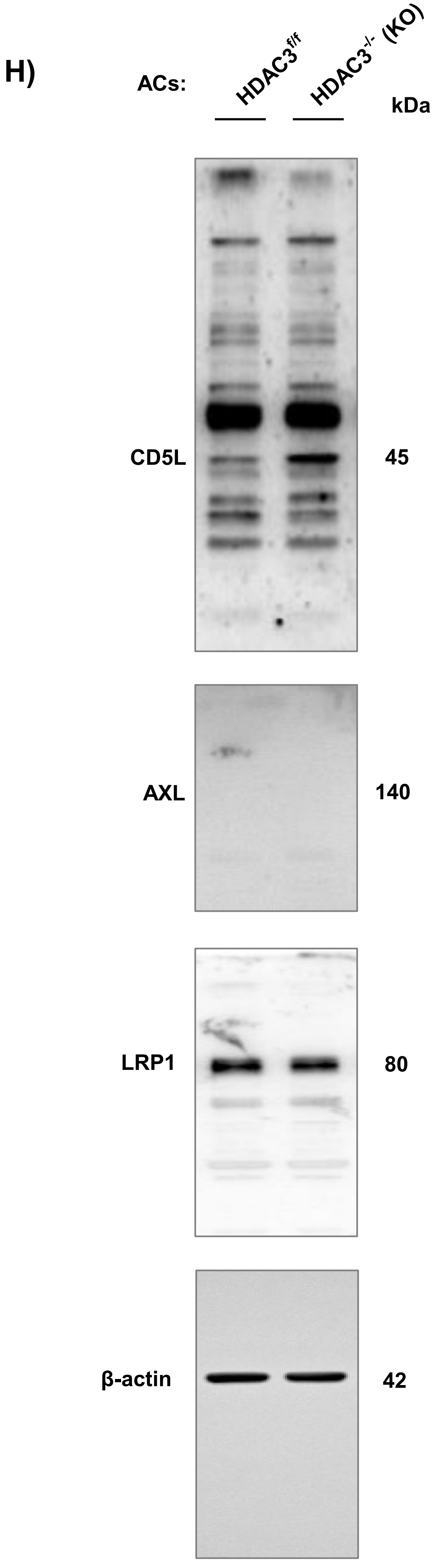

F2

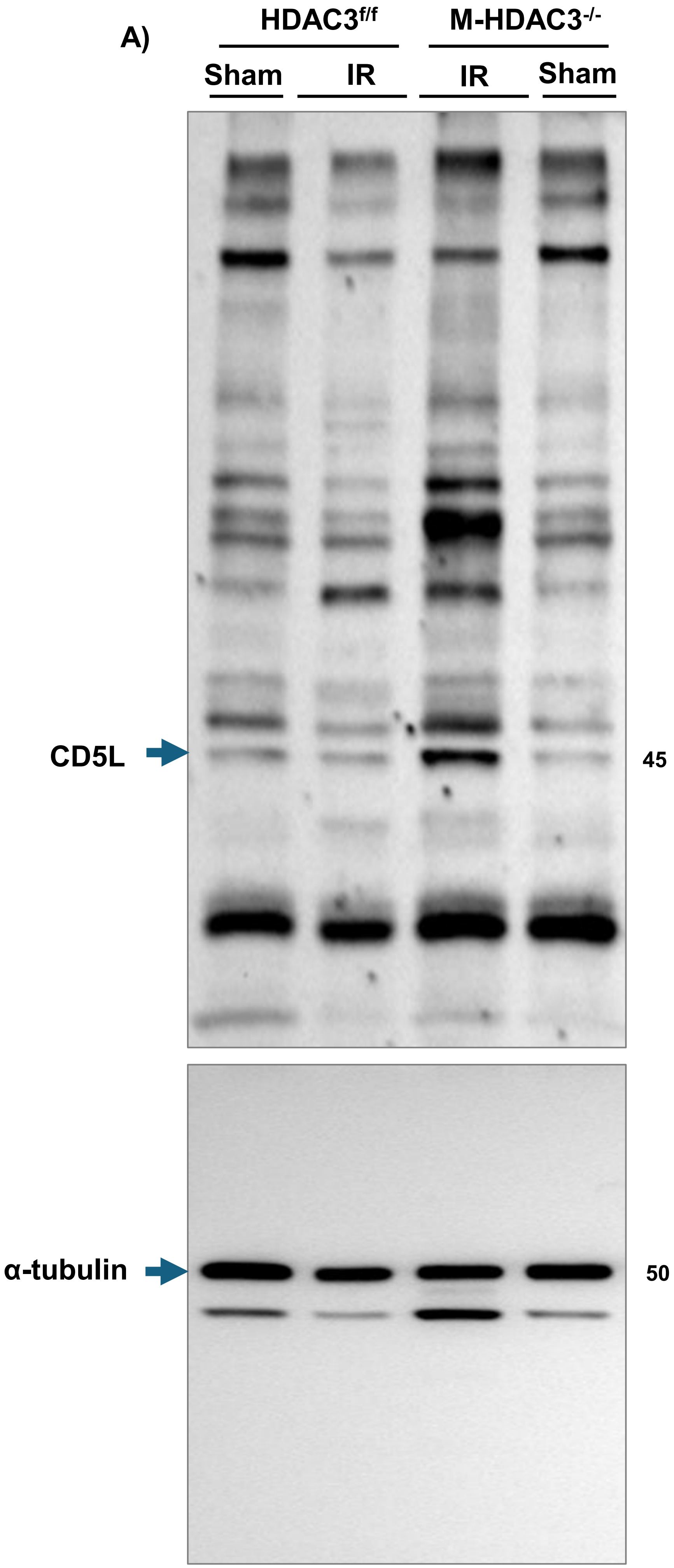

F3

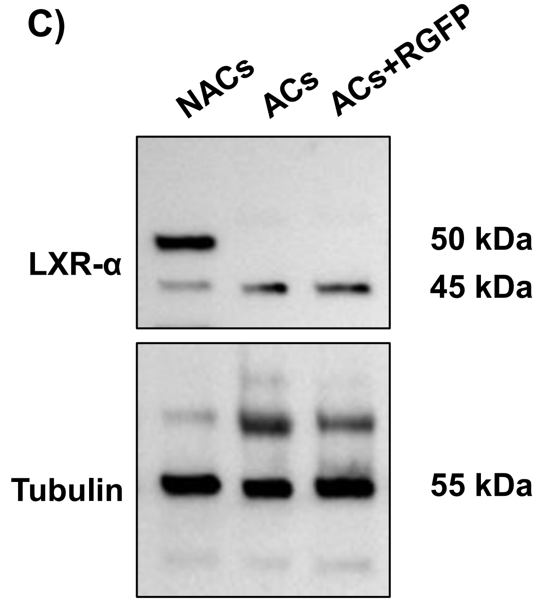
C)
NACs
ACs
ACs+RGFP
LXR-α
50 kDa
45 kDa
Tubulin
55 kDa

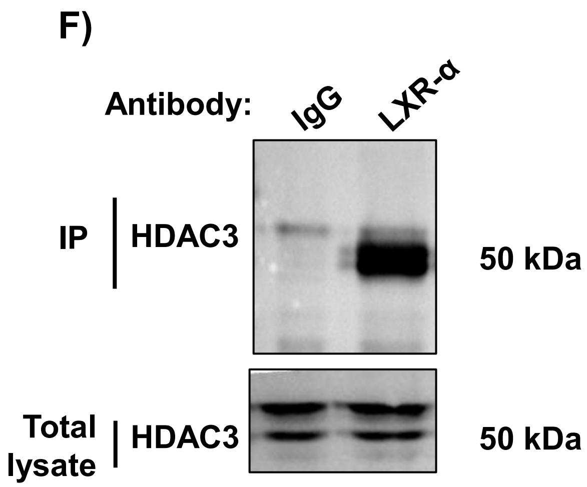
F)
Antibody:
IgG
LXR-α
IP
HDAC3
50 kDa
Total lysate
HDAC3
50 kDa

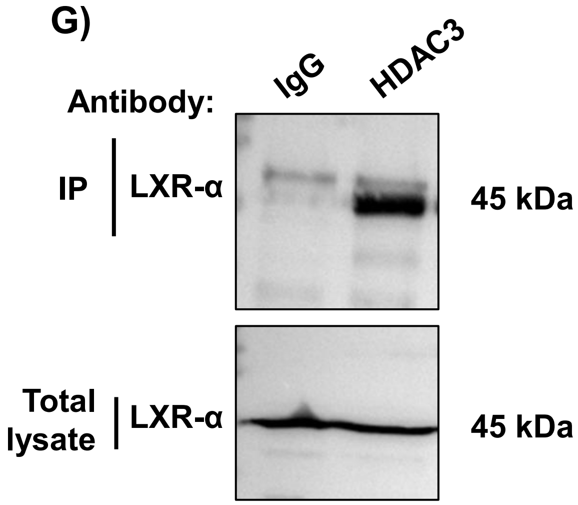
G)
Antibody:
IgG
HDAC3
IP
LXR-α
45 kDa
Total lysate
LXR-α
45 kDa

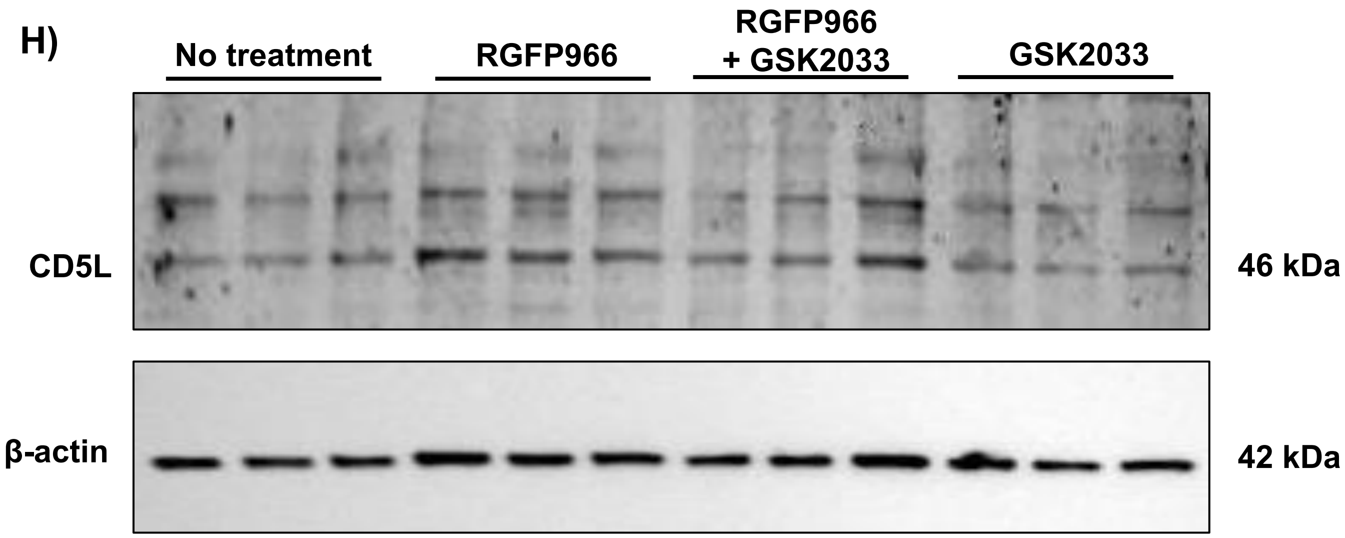
H)
No treatment
RGFP966
RGFP966
+ GSK2033
GSK2033
CD5L
46 kDa
β-actin
42 kDa

F4

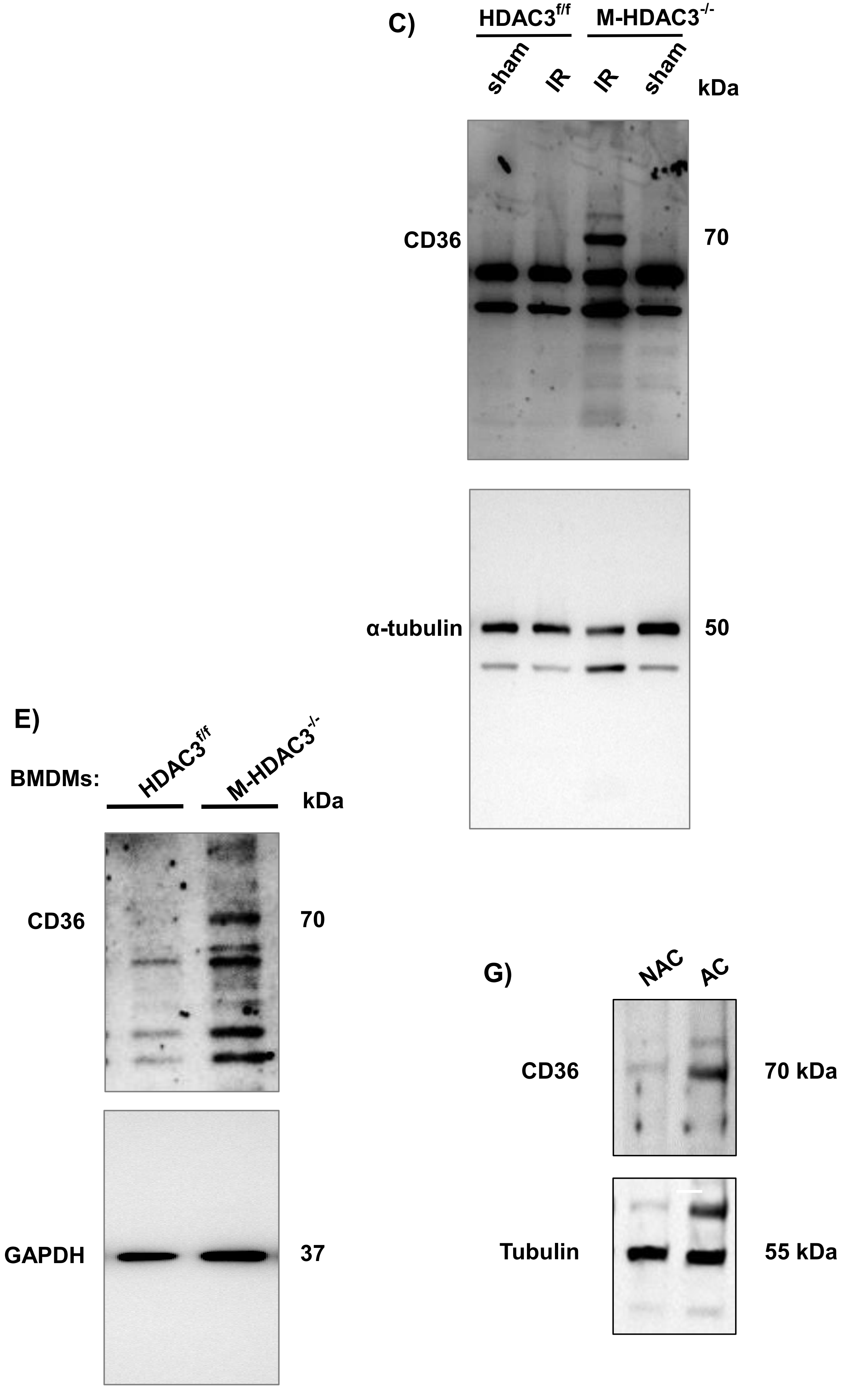
C)
HDAC3f/f
M-HDAC3-/-
sham
IR
IR
sham
kDa
CD36
70
α-tubulin
50
E)
BMDMs:
HDAC3f/f
M-HDAC3-/-
kDa
CD36
70
GAPDH
37
G)
NAC
AC
CD36
70 kDa
Tubulin
55 kDa

F5

C)

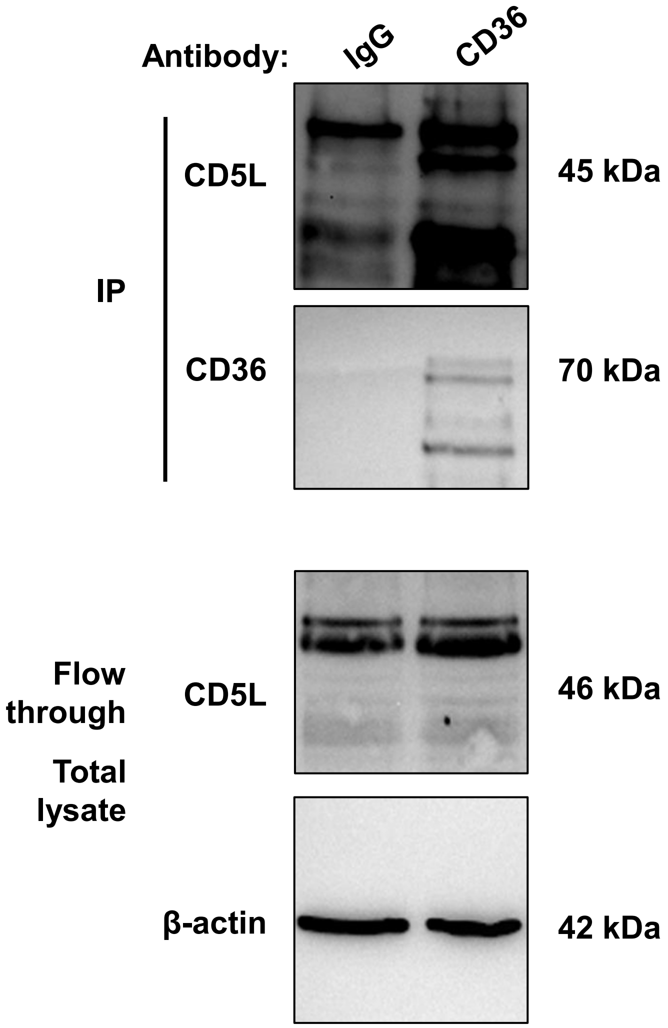
Antibody:
IgG
CD36
IP
CD5L
45 kDa
CD36
70 kDa
Flow through
CD5L
46 kDa
Total lysate
β-actin
42 kDa

F6

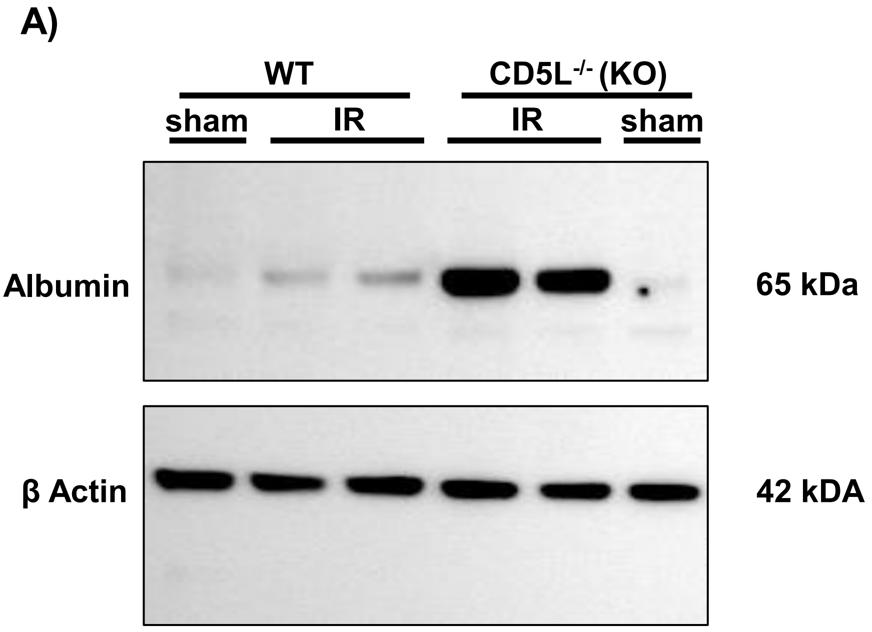

Supplement: Supplementary file 1 — Original data [file 41419_2026_8752_MOESM1_ESM.pdf]
