# Supplementary material for: CD5L promotes efferocytosis and resolution of retinal ischemic injury
Source: Cell Death Dis. 2026 Apr 20;17(1):520. doi: 10.1038/s41419-026-08752-8 (PMC13222871; doi:10.1038/s41419-026-08752-8)
Supplement: Supplementary file 2 — Supplementary data [file 41419_2026_8752_MOESM2_ESM.pdf]

Supplementary figures for the manuscript titled 'CD5L Promotes Efferocytosis and Resolution of Retinal Ischemic Injury' by Shahror *et al*.

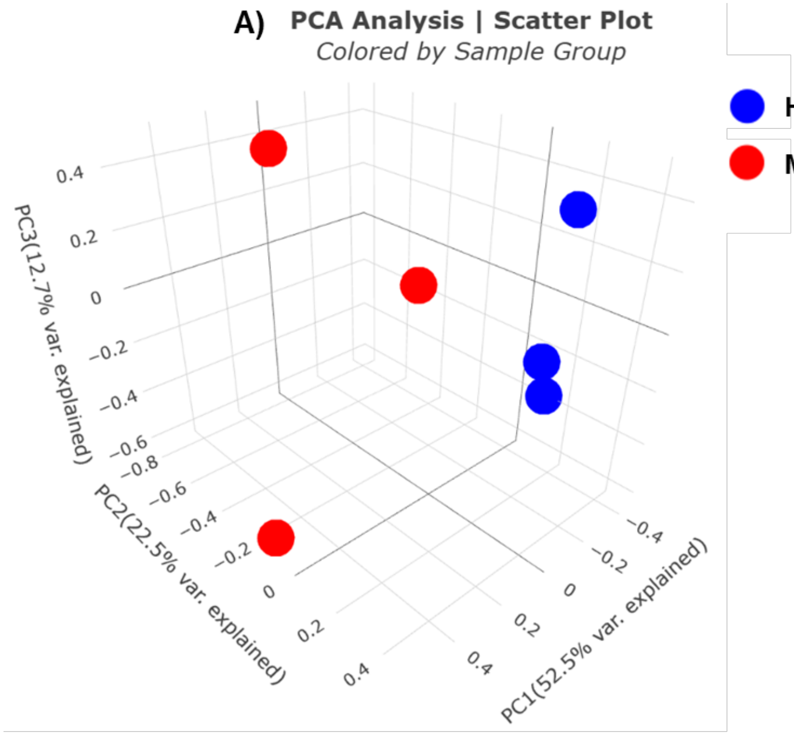

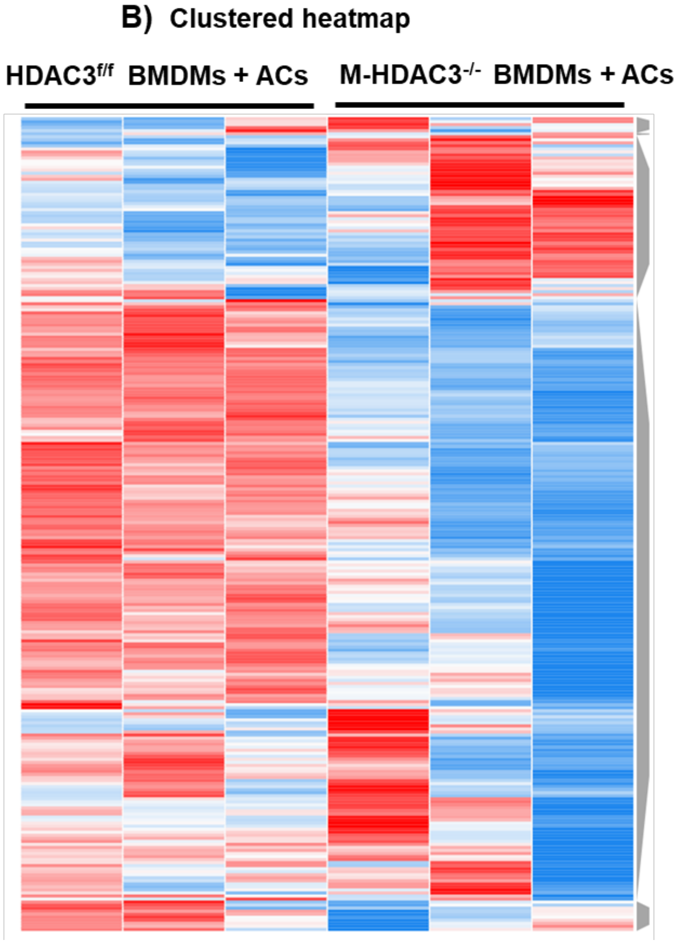

C)

Qiagen IPA BioProfiler results showing efferocytosis-related genes

Symbol	Molecule Type	Entrez Gene Name
ANXA1	enzyme	annexin A1
AXL	kinase	AXL receptor tyrosine kinase
CD36	transmembrane receptor	cluster of differentiation 36
CD47	transmembrane receptor	cluster of differentiation 47
GAS6	growth factor	growth arrest specific 6
HMGB1	transcription regulator	high mobility group box 1
IL4	cytokine	interleukin 4
ITGAV	transmembrane receptor	integrin subunit alpha V
ITGB3	transmembrane receptor	integrin subunit beta 3
JMJD6	enzyme	jumonji domain containing 6, arginine demethylase and lysine hydroxylase
LGALS3	other	galectin 3
LRP1	transmembrane receptor	LDL receptor related protein 1
MARCO	transmembrane receptor	macrophage receptor with collagenous structure
MERTK	kinase	MER proto-oncogene, tyrosine kinase
MFGE8	other	milk fat globule EGF and factor V/VIII domain containing
NR1H3 (LXRα)	ligand-dependent nuclear receptor	nuclear receptor subfamily 1 group H member 3
PFKFB3	enzyme	6-phosphofructo-2-kinase/fructose-2,6-biphosphatase3
PLAUR	transmembrane receptor	plasminogen activator, urokinase receptor
RAC1	enzyme	Rac family small GTPase 1
RARA	ligand-dependent nuclear receptor	retinoic acid receptor alpha
TGM2	enzyme	transglutaminase 2
TNF	cytokine	tumor necrosis factor
TREM2	transmembrane receptor	triggering receptor expressed on myeloid cells 2
TYROBP	transmembrane receptor	transmembrane immune signaling adaptor TYROBP

Figure S1. RNA-Seq data processing of efferocytic macrophages lacking HDAC3. A) Principal component analysis (PCA) performed using normalized RNA-seq data. Three-dimensional scatter plot of the first three Principal Components (PCs) of the data shown in the graph on the x-, y-, and z-axes. Each point represents an RNA-seq sample of HDAC3$^{-/-}$ and HDAC3$^{f/f}$ BMDMs incubated with apoptotic cells. Samples with similar gene expression profiles are closer in the three-dimensional space. **B)** Heatmaps of normalized expression values of differentially expressed genes (DEG). **C)** Table lists the efferocytosis-related genes identified using the BioProfiler analysis. Figures (A) and (B) were created using the Maayan lab https://maayanlab.cloud/biojupies/ whereas figure (C) was created using the Qiagen IPA BioProfiler.

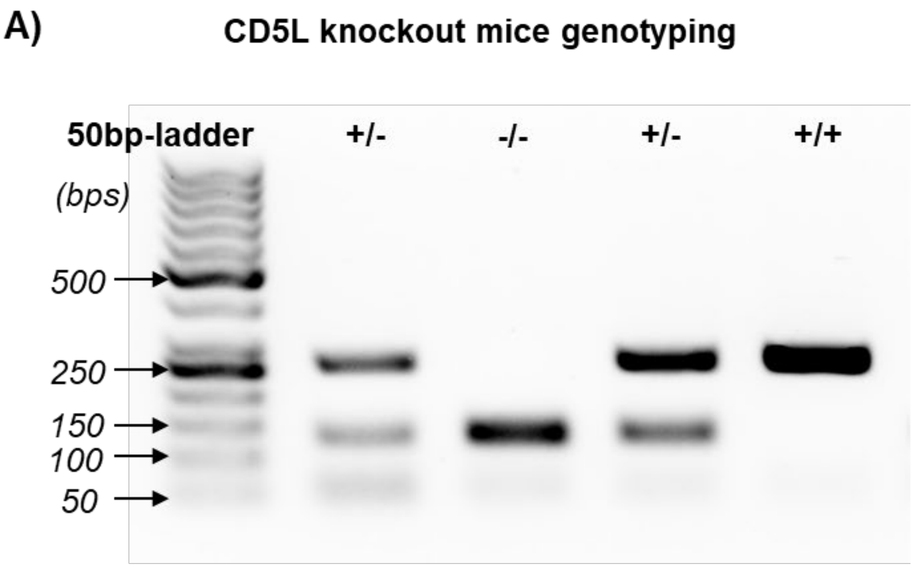

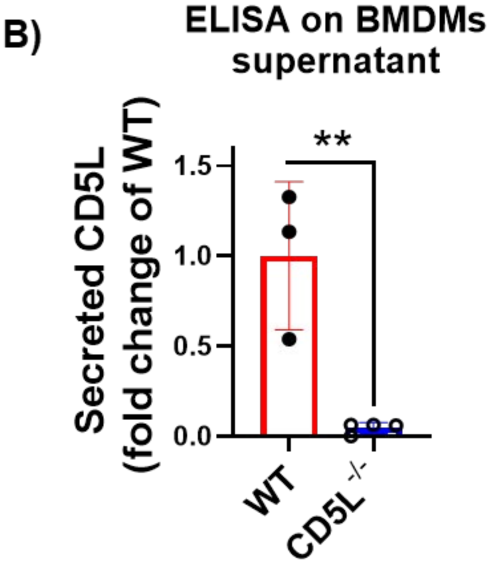

Figure S2. Characterization of CD5L knockout (CD5L KO) mice. A) Representative gel image of PCR-based genotyping of CD5L KO (CD5L$^{-/-}$) mice. The wild-type (WT) allele produces a 258 bp product, whereas the knockout (KO) allele produces a 129 bp fragment. **B)** CD5L deletion was confirmed by ELISA analysis of BMDMs supernatant, demonstrating the absence of CD5L secreted protein in the CD5L$^{-/-}$ BMDMs media compared to WT BMDM, n=3-4, **p<0.01.

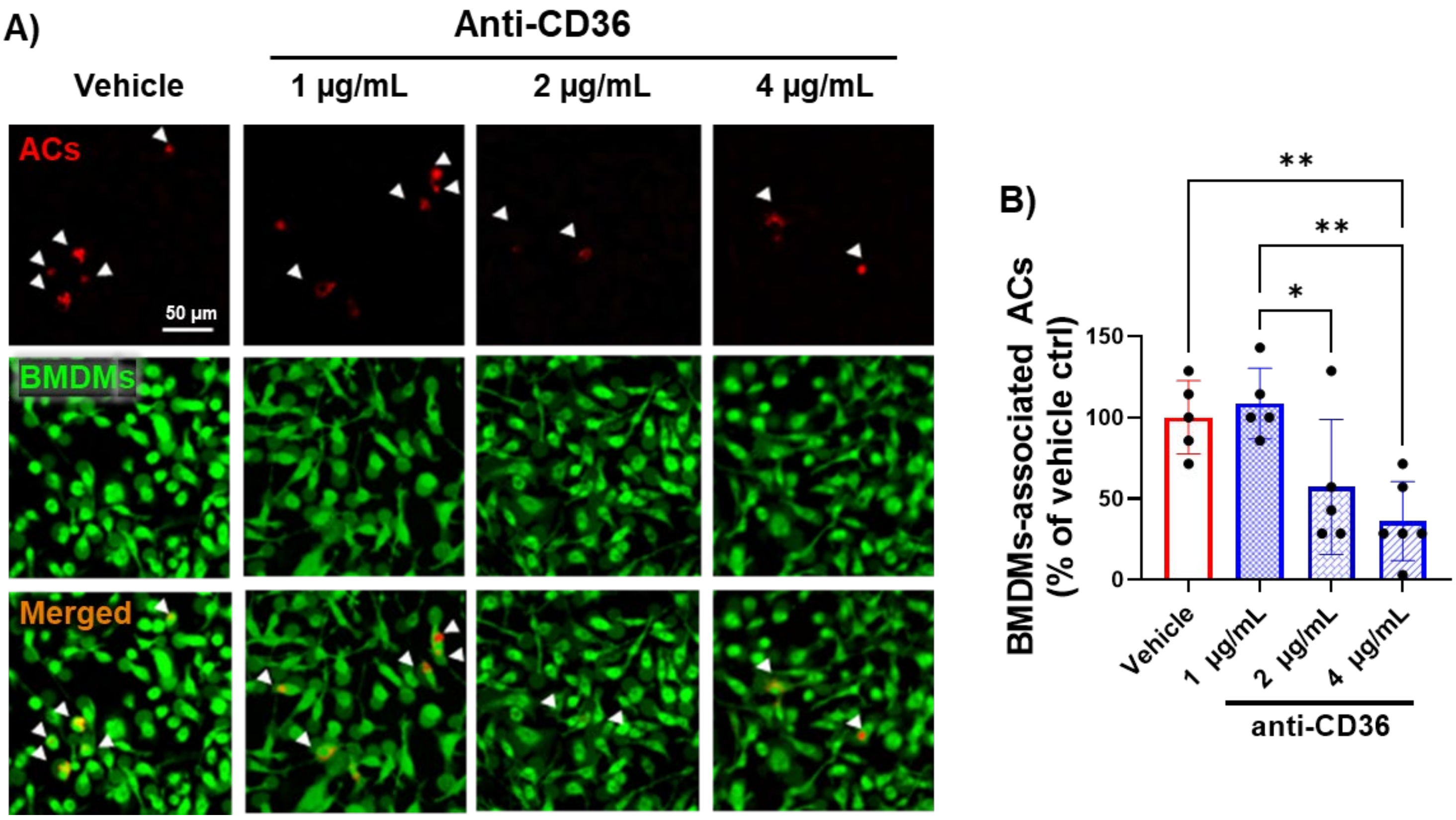

Figure S3. CD36 neutralization decreases macrophage efferocytosis in vitro. A) Representative images of an in vitro efferocytosis assay where CM-DiI red-labeled ACs were co-cultured with CFDA green-labeled WT BMDMs that were pretreated with increasing concentrations of CD36 neutralizing antibody. **B)** Quantification showed CD36 neutralizing antibody inhibiting efferocytosis in a concentration-dependent manner, n=5-6, *p<0.05, **p<0.01.

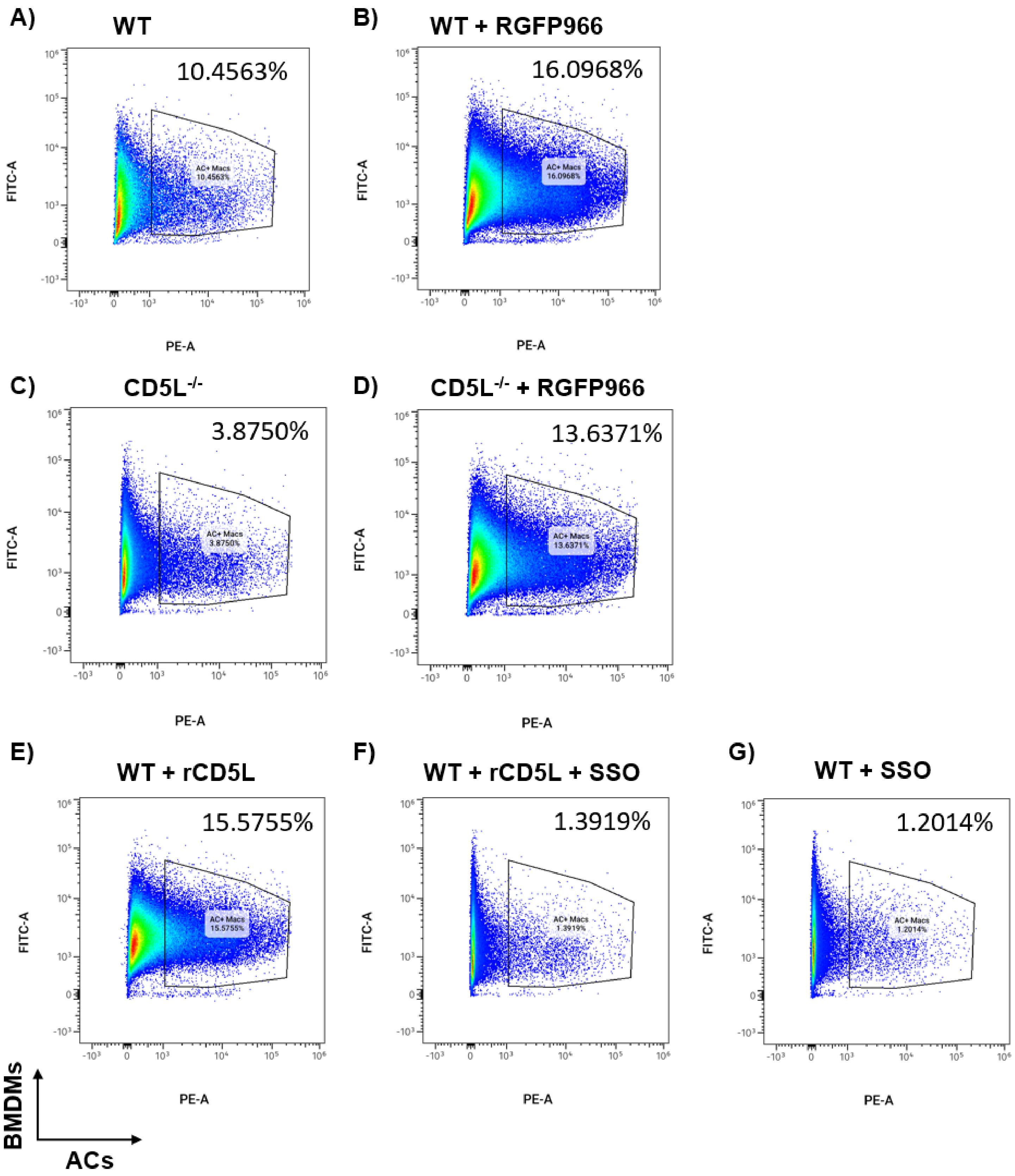

Figure S4. Representative flow cytometric confirmation of in vitro efferocytosis assay imaging data (figures 3A, 5D in the original manuscript). CFDA green-labeled WT and CD5L$^{-/-}$ BMDMs were treated with

RGFP966 (2 μM), rCD5L (1 μg/ml), or SSO (200 μM) and incubated with CM-DiI red-labeled ACs, followed by washing of unengulfed ACs and suspension of BMDMs for flowcytometric analysis of macrophages that are positive for ACs (denoting engulfment).

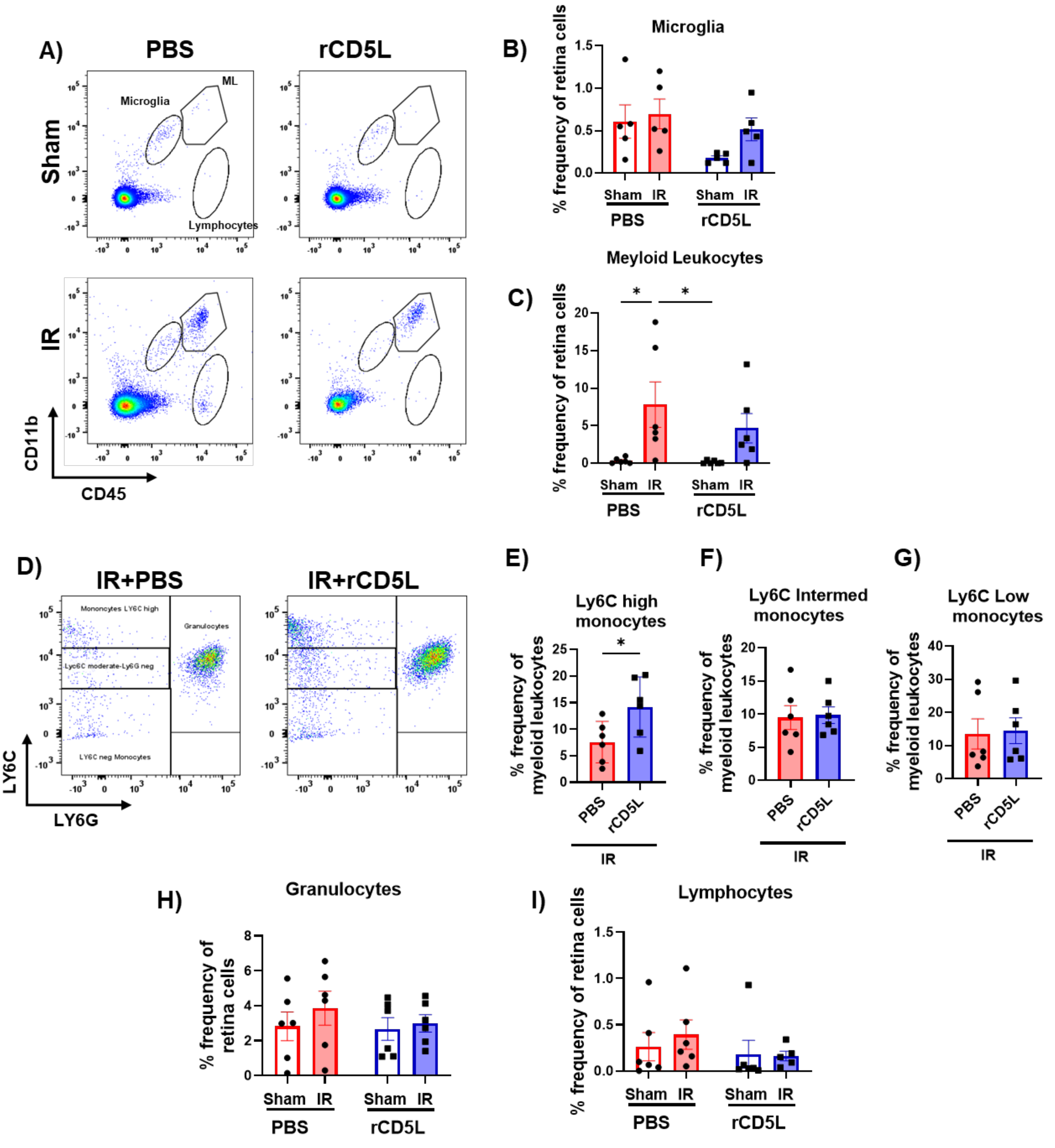

Figure S5. Effect of rCD5L treatment on IR-induced microglia proliferation and myeloid leukocyte infiltration into the WT retina at 48 hours after injury. A) Representative graphs of the flow-cytometry analyses gating strategy that was used to quantify the immune cell populations of microglia, myeloid leukocytes (ML), and lymphocytes in WT retinas treated with rCD5L (1μg/eye) or PBS (control). **B, C)** The percentage of

microglia and ML was not different between the IR groups. **D)** Representative scatter blots showing the gating strategy of ML based on Ly6C/Ly6G expression into Ly6C^{high}, Ly6C^{intermediate}, Ly6C^{negative} monocytes that were negative for Ly6G, and Ly6C^{+}/Ly6G^{+} granulocytes. **E-G)**The percentage of Ly6C^{hi} monocytes was found to be elevated in rCD5L-treated injured retinas compared to control treatment, with no change in Ly6C^{intermediate}, Ly6C^{neg} monocytes population between the IR groups. **H, I)** Quantification of granulocytes and lymphocytes, respectively, showed no change between the groups. For each data point, three retinas were pooled, and n=4-5 preparations of pooled retinas were analyzed per group, *p<0.05.
